# MIR497HG‐Derived miR‐195 and miR‐497 Mediate Tamoxifen Resistance via PI3K/AKT Signaling in Breast Cancer

**DOI:** 10.1002/advs.202204819

**Published:** 2023-02-23

**Authors:** Yao Tian, Zhao‐Hui Chen, Peng Wu, Di Zhang, Yue Ma, Xiao‐Feng Liu, Xin Wang, Dan Ding, Xu‐Chen Cao, Yue Yu

**Affiliations:** ^1^ The First Department of Breast Cancer Tianjin Medical University Cancer Institute and Hospital National Clinical Research Center for Cancer Tianjin 300060 China; ^2^ Key Laboratory of Cancer Prevention and Therapy Tianjin 300060 China; ^3^ Tianjin's Clinical Research Center for Cancer Tianjin 300060 China; ^4^ Key Laboratory of Breast Cancer Prevention and Therapy Tianjin Medical University Ministry of Education Tianjin 300060 China; ^5^ Department of General Surgery Tianjin Medical University General Hospital Tianjin 300052 China; ^6^ State Key Laboratory of Medicinal Chemical Biology Key Laboratory of Bioactive Materials Ministry of Education and College of Life Sciences Nankai University Tianjin 300071 China

**Keywords:** breast cancer, miR‐497/195, MIR497HG, PI3K‐AKT, tamoxifen resistance

## Abstract

Tamoxifen is commonly used for the treatment of patients with estrogen receptor‐positive (ER+) breast cancer, but the acquired resistance to tamoxifen presents a critical challenge of breast cancer therapeutics. Recently, long noncoding RNA MIR497HG and its embedded miR‐497 and miR‐195 are proved to play significant roles in many types of human cancers, but their roles in tamoxifen‐resistant breast cancer remain unknown. The results indicate that MIR497HG deficiency induces breast cancer progression and tamoxifen resistance by inducing downregulation of miR‐497/195. miR‐497/195 coordinately represses five positive PI3K‐AKT regulators (MAP2K1, AKT3, BCL2, RAF1, and CCND1), resulting in inhibition of PI3K‐AKT signaling, and PI3K‐AKT inhibition in tamoxifen‐resistant cells restored tamoxifen responsiveness. Furthermore, ER *α* binds the MIR497HG promoter to activate its transcription in an estrogen‐dependent manner. ZEB1 interacts with HDAC1/2 and DNMT3B at the MIR497HG promoter, resulting in promoter hypermethylation and histone deacetylation. The findings reveal that ZEB1‐induced MIR497HG depletion contributes to breast cancer progression and tamoxifen resistance through PI3K‐AKT signaling. MIR497HG can be used as a biomarker for predicting tamoxifen sensitivity in patients with ER+ breast cancer.

## Graphical abstract

This work presents a novel mechanism underlying tamoxifen resistance caused by ZEB1‐mediated histone deacetylation and DNA methylation of the MIR497HG promoter. The derivatives of MIR497HG, miR‐497, and miR‐195, work together to inhibit PI3K‐AKT signaling by downregulating the five positive regulators of PI3K‐AKT signaling, indicating MIR497HG can serve as a prognostic factor for tamoxifen sensitivity in patients with ER+ breast cancer.

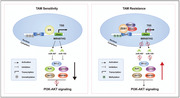

## 1 Introduction

Breast cancer has surpassed lung cancer in terms of new cases, becoming the most extensively diagnosed cancer in 2020 and the fifth chief cause of cancer death.^[^ ^1^ ^]^ Nearly 70% of breast cancer patients are estrogen receptor‐positive (ER+), making them candidates for endocrine therapies such as tamoxifen, fulvestrant, and letrozole.^[^ ^2^ ^]^ Tamoxifen, a selective ER modulator, binds to ER competitively and inhibits ER‐induced breast cancer cell growth. As an effective antiestrogenic drug, tamoxifen is extensively used as standard therapy for ER+ breast cancer patients. However, tamoxifen resistance acquisition becomes a significant barrier during endocrine therapy.^[^ ^3^ ^]^ Several biological tamoxifen‐resistant mechanisms have been reported, including estrogen receptor 1(ESR1) mutation/deletion and other cell proliferation pathways activation, among which, phosphatidylinositol‐3‐kinase (PI3K)‐AKT signaling has been studied most extensively and thoroughly.^[^ ^4^, ^5^, ^6^ ^]^ PI3K‐AKT signaling is reported to be often activated in endocrine‐resistant breast cancer and established an important cross talk with ER.^[^ ^7^ ^]^ However, in‐depth understanding of further mechanisms of tamoxifen resistance remains critical.

Noncoding RNAs (ncRNAs), particularly microRNAs (miRNAs) and long noncoding RNAs (lncRNAs), exhibit significant functions in epigenetic regulation of target genes and are involved in normal cellular function and human disease, including cancer.^[^ ^8^ ^]^ Mounting evidence shows that abnormally expressed ncRNAs are involved in many human cancers and always act as essential tumor‐suppressor genes or oncogenes to regulate breast tumorigenesis and progression.^[^ ^9^, ^10^ ^]^ A complex interplay between miRNAs and lncRNAs has been identified, with some lncRNAs thought to be host genes for miRNAs and processed to produce miRNAs, suppressing target genes.^[^ ^11^, ^12^ ^]^ Studies have shown that lncRNA MIR497HG, located on chromosome 17p, is decreased and function as a tumor suppressor in bladder cancer.^[^ ^13^, ^14^ ^]^ Increasing evidence suggests that MIR497HG‐derived miR‐497/195 cluster is decreased and become tumor suppressors in various kinds of human malignances, including hepatocellular carcinoma,^[^ ^15^, ^16^ ^]^ bladder cancer,^[^ ^13^, ^17^ ^]^ lung cancer,^[^ ^18^, ^19^ ^]^ glioma,^[^ ^20^ ^]^ colon cancer,^[^ ^21^ ^]^ ovarian cancer,^[^ ^22^ ^]^ acute lymphoblastic leukemia,^[^ ^23^ ^]^ and breast cancer.^[^ ^24^, ^25^ ^]^ However, it is unclear whether MIR497HG or its derivatives miR‐497/195 were correlated to the process of acquired resistance to tamoxifen during breast cancer progression.

In this study, we found a significant function for lncRNA MIR497HG and its embedded miRNAs, miR‐195, and miR‐497, in conferring tamoxifen resistance in breast cancer. The analysis demonstrated that decreased miR‐497/195 or MIR497HG expression in breast cancer can promote aggressiveness, estrogen‐independent proliferation, and tamoxifen resistance. miR‐497 and miR‐195 coordinately downregulated five positive regulators of PI3K‐AKT signaling (MAP2K1, AKT3, BCL2, RAF1, and CCND1), resulting in PI3K‐AKT signaling pathway activation. Inhibiting PI3K‐AKT signaling restored tamoxifen responsiveness in vitro and in vivo. Additionally, ER *α* could transactivate MIR497HG and miR‐497/195 in an estrogen‐dependent manner. DNA methylation or histone deacetylation of MIR497HG was the primary suppressor of MIR497HG and miR‐497/195 expression. This work also revealed that recruiting DNMT3B or HDAC1/2 resulted in ZEB1‐dependent suppression of MIR497HG. To the best of our knowledge, this research is the first to identify the epigenetic regulation of MIR497HG in tamoxifen resistance. The findings present a promising and representative method of identifying biomarkers for treating endocrine resistance in breast cancer.

## 2 Results

### 2.1 Depletion of miR‐497/195 Cluster and Their Host Gene MIR497HG Are Related to Poor Outcome of Breast Cancer Patients

To investigate transcriptional levels of 25 known miRNAs on chromosome 17p, we examined the copy‐number variation of these miRNAs in the TCGA database. As demonstrated in Figure S1 of the Supporting Information, the miR‐497/195 cluster was the most frequently deleted miRNA genes in TCGA breast cancer specimens (651/1070, 60.8%). Compared to normal breast specimens, the expression of miR‐195/497 was remarkably reduced in breast cancer specimen (Figure S1B,C, Supporting Information). Their host gene, MIR497HG, was also downregulated in breast cancer sample (Figure S1D, Supporting Information). Besides, the expression levels of other miRNAs were exhibited in Figure S2 of the Supporting Information. Next, the KM‐plotter was used to compare the overall survival of patients who expressed different levels of miR‐195, miR‐497, or MIR497HG. We found that patients with low miR‐195, miR‐497, or MIR497HG levels exhibited a remarkably worse prognosis than those with high miR‐195, miR‐497, or MIR497HG levels (Figure S1E–G, Supporting Information). Moreover, MIR497HG expression was positively correlation with miR‐497 (Figure S1H, Supporting Information) and miR‐195 (Figure S1I, Supporting Information). These findings imply that depletion of miR‐497/195 cluster and their host gene MIR497HG is correlated with a worse prognosis in breast cancer.

### 2.2 MIR497HG Promoter Methylation Regulates the Expression of miR‐497/195 and MIR497HG

**Figure 1:**
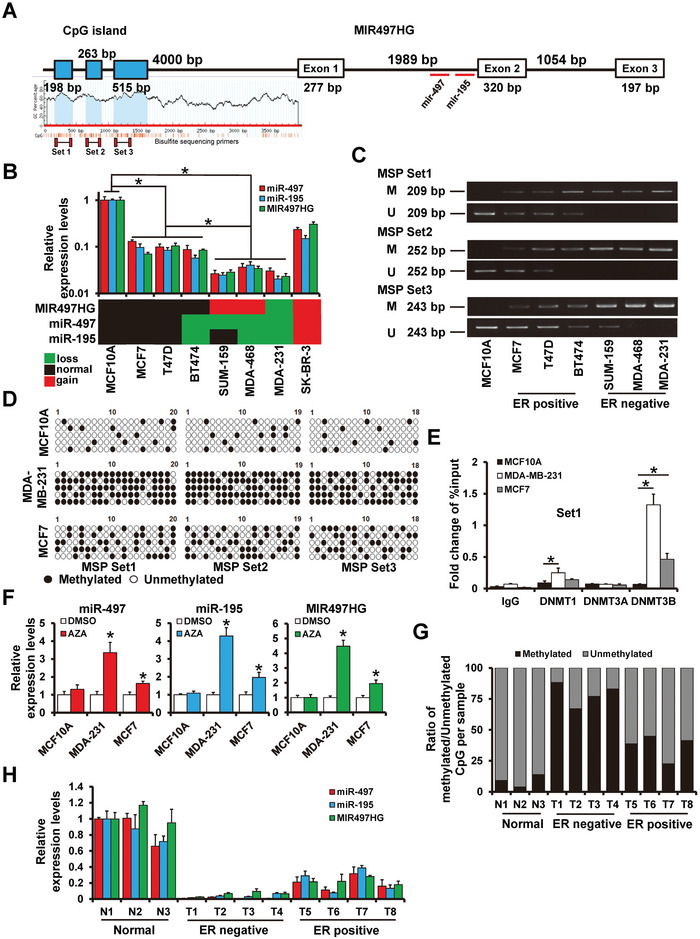
miR‐497/195 and its host gene MIR497HG are epigenetically regulated in breast cancer. A) Schematic illustration of MIR497HG's genomic structure. miR‐497/195 that maps within intron 1 of MIR497HG is shown using a red horizontal bar. B) RT‐qPCR results showing expression levels of MIR497HG and miR‐497/195 in breast cancer cell lines. C) Methylation specific PCR of bisulfite‐modified DNA in normal breast epithelial cell line and breast cancerous cell lines. D) Bisulfite sequence data for MCF10A, MDA‐MB‐231, and MCF7. E) Enrichment of DNMTs on MIR497HG promoter region determined by ChIP sequence analysis. F) RT‐qPCR results showing profiles of MIR497HG and miR‐497/195 expression in indicated cell lines treated with or without AZA. G) Ratio of overall methylated over unmethylated CpGs by bisulfite sequencing in breast cancer tissues with different molecular subtyping relative to normal tissues. H) Levels of MIR497HG and miR‐497/195 expression in breast cancer tissues with different molecular subtypes relative to normal tissues. *p < 0.05.

A schematic illustration of the miR‐497/195 and MIR497HG is shown in **Figure** **1**A. The miR‐195/497 cluster is located in the first intron of MIR497HG, and the existences of CpG islands on its promoter region were predicted by Meth Primer analysis tool. Accordingly, we hypothesized that MIR497HG is the host gene for miR‐195/497 cluster, and that promoter methylation epigenetically regulates both MIR497HG and miR‐195/497 clusters. To confirm this, reverse transcription‐quantitative polymerase chain reaction (RT‐qPCR) was employed to analyze the relationship of MIR497HG and miR‐195/497 clusters in a series of breast cancer cells lines (T47D, MCF7, BT474, SUM‐159, MDA‐MB‐231, MDA‐MB‐468, and SKBR3), normalized by the levels of normal breast cell line, MCF10A. The data showed significantly lower expression of MIR497HG and miR‐195/497 in wholly breast cancer cell lines (Figure 1B). Moreover, we found a notable discrepancy in the expression of miR‐497/195 and MIR497HG between the triple‐negative breast and hormone receptor‐positive (HR+) breast cancer cells. miR‐497/195 and MIR497HG were highly expressed in HR+ breast cancer subtypes (MCF7, T47D, and BT474), but significantly lowly expressed in triple‐negative breast cancer subtype (MDA‐MB‐231, SUM‐159, and MDA‐MB‐468) (Figure 1B). To verify the methylation of MIR497HG promoter region as a mechanism for the regulation of both MIR497HG and miR‐497/195 cluster, bisulfite sequencing (BSP) and methylation specific PCR (MSP) were employed to assess the methylation status on the MIR497HG promoter region. MSP of three different CpG island regions (Figure 1A) revealed that a remarkable PCR product was amplified from the MCF10A (normal breast epithelial cell line) and T47D, MCF7, and BT474 (HR+ breast cancer cell lines) using unmethylation‐specific primers. On the other hand, a PCR product with remarkably lower intensity was observed in SUM‐159 MDA‐MB‐468, and MDA‐MB‐231 (Figure 1C). Contrary results were observed with methylation‐specific primers (Figure 1C). Moreover, BSP was performed to confirm the methylation of MIR497HG‐associated CpG island in MCF10A, MCF7, and MDA‐MB‐231 cell lines. The proportion of methylation in the MCF10A cell line, where MIR497HG and miR‐497/195 were highly expressed, was 46/285 (16.1%). Contrarily, the methylation ratio in MCF7 and MDA‐MB‐231 cell lines expressing medium or low levels of miR‐497/195 and MIR497HG, was 121/285 (42.5%) and 219/285 (76.8%), respectively (Figure 1D). Chromatin immunoprecipitation (ChIP) analyses revealed that MDA‐MB‐231 cell line had higher methyltransferase recruitment than the MCF10A and MCF7 cell lines (Figure 1E). The expression of miR‐497/195 and MIR497HG was elevated in MDA‐MB‐231 and MCF7 cells treating with the DNA demethylating agent 5‐aza‐2‐deoxycytidine (AZA), but not in MCF10A cell line (Figure 1F). The ratio of MIR497GH promoter methylation was higher in ER− breast cancer patients (Figure 1G). Contrary to normal breast specimens, the expression of miR‐195/497 and MIR497HG was remarkably decreased in breast cancer specimens (Figure 1H). Furthermore, their expression was remarkably reduced in ER− breast cancer specimens (Figure 1H). These findings demonstrated a role for DNA methylation in underexpressed MIR497HG, miR‐195/497 in breast cancer cells.

### 2.3 MIR497HG Suppresses Breast Cancer Progression by Mediating miR‐195/497

**Figure 2:**
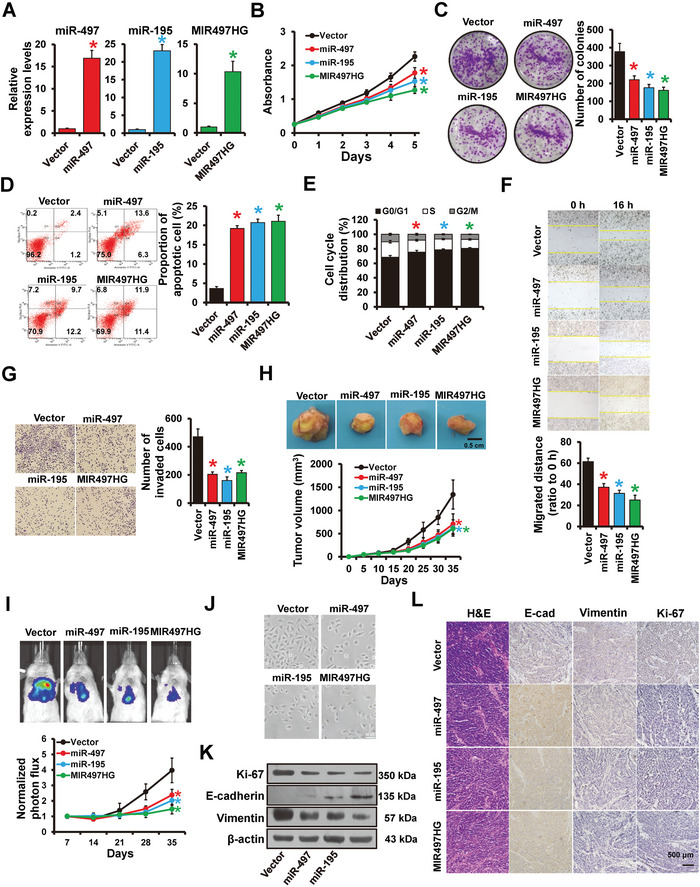
MIR497HG and miR‐497/195 inhibits breast cancer progression both in vitro and in vivo. A) The expression levels of MIR497HG, miR‐497, and miR‐195 in stable MDA‐MB‐231 cell lines detected by RT‐qPCR. B,C) Proliferation abilities of MDA‐MB‐231 cells stably transfected with plasmids containing MIR497HG, miR‐497, miR‐195, or control vector determined by (B) MTT and (C) colony formation assays. D) Cell apoptosis and E) cell cycle distribution of MDA‐MB‐231 cells transfected with MIR497HG, miR‐497, and miR‐195, or control vector determined by flow cytometry. F) Migration abilities of MDA‐MB‐231 cells with different transfections were analyzed by wound‐healing assay. G) Invasion abilities of MDA‐MB‐231 cells with different transfections were determined by Transwell assays. H) Tumor growth curves of subcutaneous tumors made by MDA‐MB‐231 cells with overexpression of MIR497HG or miR‐497/195, as well as control vector cells at the indicated times. Representative photos of the tumors at the harvest time are also shown. I) Tail vein metastasis in mice injected with MDA‐MB‐231 cells with forced expression of MIR497HG or miR‐497/195, as well as control vector cells, determined via living imaging and quantification plot. J) Morphology of MDA‐MB‐231 cells with forced expression of MIR497HG or miR‐497/195, as well as control vector cells. K) Western blots showing expression levels of mesenchymal marker Vimentin, epithelial marker E‐cadherin and Ki‐67 proteins in MDA‐MB‐231 cells expressing MIR497HG, miR‐497/195, or control vector cells. L) Immunohistochemical staining images showing expression of Vimentin, E‐cadherin and Ki‐67 in MIR497HG‐ or miR‐497/195‐overexpressed MDA‐MB‐231 and control subcutaneous tumors. *P < 0.05.

To investigate the functions of miR‐195/497 and MIR497HG in breast cancer progression, we constructed stable MIR497HG (231‐MIR497HG)‐, miR‐497 (231‐miR‐497)‐ or miR‐195 (231‐miR‐195)‐overexpressed cells and control cells (231‐Vector) using MDA‐MB‐231 cell line via lentiviral infection (**Figure** **2**A). Forced expression of miR‐497, miR‐195, or MIR497HG could reduce cell viability and colony formation in vitro (Figure 2B,C). Flow cytometry analyses showed that forced expression of MIR497HG, miR‐497, or miR‐195 upregulated the percentage of apoptotic cells (Figure 2D) and caused arrest at the G1 phases of cell cycle (Figure 2E). The consistent results were obtained from MCF7‐MIR497HG‐, MCF7‐miR‐497‐, and MCF7‐miR‐195‐overpressed cells and control cells (Figure S3A–D, Supporting Information). Moreover, wound‐healing (Figure 2F) and transwell (Figure 2G) assays demonstrated that exogenous overexpression of MIR497HG, miR‐497, or miR‐195 can suppress the cell migration and invasion. To learn more about the effects of MIR497HG and miR‐497/195 on breast cancer progression in vivo, 231‐Vector, 231‐MIR497HG, 231‐miR‐497, and 231‐miR‐195 cells were injected subcutaneously (Figure 2H) or intravenously (Figure 2I), followed by the assessment of metastasis and tumor growth at 35 days postinjection. There was a significant reduction in orthotopic tumor size and lung metastasis in the MIR497HG‐, miR‐497‐ or miR‐195‐overexpression groups compared to the control group. Epithelial to mesenchymal transition (EMT) is a driver of cancer metastasis and contributes to cancer cell invasion and migration.^[^ ^26^ ^]^ The present analysis indicated that forced expression of miR‐195, or miR‐497, or MIR497HG in the mesenchymal MDA‐MB‐231 resulted in epithelial phenotypic conversion (Figure 2J). Forced expression of miR‐195, miR‐497 or MIR497HG in MDA‐MB‐231 cells consistently elevated the E‐cadherin expression (epithelial marker) while decreasing the Vimentin expression (mesenchymal marker) compared to control cells (Figure 2K). Ki‐67 expression was also downregulated in MDA‐MB‐231 cells that overexpressed MIR497HG, miR‐497, or miR‐195 (Figure 2K). Immunohistochemical staining revealed that tumors from 231‐MIR497HG, 231‐miR‐497, or 231‐miR‐195 mice had higher levels of E‐cadherin but lower levels of Vimentin and Ki‐67 when compared to those from 231‐Vector mice (Figure 2L). These findings indicate that MIR497HG and miR‐497/195 act as tumor suppressors in breast cancer.

Because lncRNAs rely heavily on their derived miRNAs, we investigated whether the MIR497HG‐associated phenotypes in breast cancer are mediated by miR‐497/195. MIR497HG knockdown remarkably reduced miR‐497/195 expression in T47D cells (Figure S4A, Supporting Information). Next, miR‐195 or miR‐497 mimics were cotransfected into T47D cells with shMIR497HG. The promotion of cell proliferation, invasion, migration, and EMT‐like phenotype induced by MIR497HG depletion was significantly reversed by miR‐195/497 overexpression (Figure S4B–H, Supporting Information). Consistently, the tumor repression of MIR497HG on MCF7 cells could be reversed by anti‐miR‐497 and anti‐miR‐195 (Figure S3A–D, Supporting Information). These results indicate that miR‐195 and miR‐497 are crucial MIR497HG derivatives in breast cancer progression.

### 2.4 ER*α* Transactivates MIR497HG Expression

**Figure 3:**
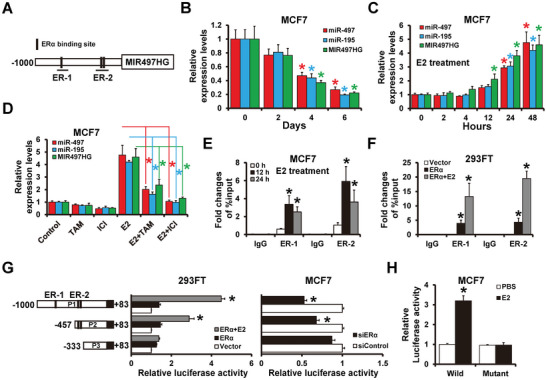
ER*α* transactivates MIR497HG expression. A) Schematic illustration of MIR497HG's promoter region (−1000 to +1). Horizontal black bars at the bottom denote the amplicons used in ChIP sequence analysis. B) RT‐qPCR results showing levels of MIR497HG and miR‐497/195 expression in hormone‐deprived MCF7 cells. C) RT‐qPCR results showing levels of MIR497HG expression in hormone‐deprived MCF7 cells after treatment with E2 (10 nm). D) Levels of MIR497HG and miR‐497/195 expression in hormone‐deprived MCF7 cells after 48 h of treatment with E2 (10 nm) and/or tamoxifen/fulvestrant (1 µm). E,F) Enrichment of ER*α* on the MIR497HG promoter region in hormone‐deprived MCF7 (E) and 293FT (F) cells determined by ChIP analysis. MCF7 cells were subsequently treated with 10 nm E2 for 12 or 24 h, while 293FT cells were transfected with an ER*α*‐expressing plasmid with or without 10 nm E2 treatment for 24 h. Enrichment of MIR497HG promoter DNA in the ER*α*‐immunoprecipitated samples was subsequently determined by qPCR. G) Dual‐luciferase reporter assay results showing regulation of MIR497HG promoter activity by ER*α*. Several luciferase reporter plasmids, containing different deleted MIR497HG promoter regions, were transfected with an ER*α*‐expressing plasmid into 293FT cells (left) or with ER*α* siRNAs into MCF7 cells (right). H) Regulation of ER*α*‐binding site‐mutated MIR497HG promoter activity by E2 as determined by dual‐luciferase reporter assay. MCF7 cells cultured in hormone‐deprived condition were transfected with wild‐type or mutant MIR497HG promoter luciferase reporter plasmids, then treated with 10 nm E2 for 48 h. *p < 0.05.

As previously stated, MIR497HG and miR‐497/195 were remarkably highly expressed in ER+ breast cancer cells compared to ER− breast cancer cells. Therefore, we attempted to ascertain whether ER*α* regulates MIR497HG. Three ER*α* binding sites were identified on the MIR497HG promoter (**Figure** **3**A). Expressions of MIR497HG and miR‐497/195 in estrogen‐deficient media was notably decreased in a time‐dependent manner (Figure 3B), whereas E2 treatment significantly increased expressions of MIR497HG and miR‐497/195 in MCF7 cells (Figure 3C). Furthermore, estrogen‐stimulated expression of MIR497HG and miR‐497/195 was significantly decreased in response to the estrogen receptor *α* antagonists, tamoxifen or fulvestrant (Figure 3D). The ChIP assay was then used to immunoprecipitate ER*α* in MCF7 and 293FT cells. We found ER*α* occupancy on both ER‐1 and ER‐2 of the MIR497HG promoter region in MCF7 cells (Figure 3E) and ER*α*‐transfected 293FT cells (Figure 3F) after E2 treatment. To see ER*α* regulates MIR497HG promoter activity, several deletion mutants of MIR497HG promoter region with or without ER*α* binding sites were cloned into a pGL3‐basic reporter (left panel of Figure 3G). Following the transfection of the reporter into 293FT or MCF7 cells, the promoter activity was measured using luciferase assays. Exogenous ER*α* expression in 293FT (ER‐) cells remarkably increased luciferase activity in constructs containing ER*α* binding sites (P1 and P2) but not in construct P3 (middle panel of Figure 3G; middle). On the other hand, luciferase reporter activities in MCF7 cells were significantly reduced after transfection with ER*α*‐targeting siRNA (right panel of Figure 3G). Furthermore, the mutation of the ER*α*‐binding site eliminated E2 reactivity to the MIR497HG promoter (Figure 3H). Analogous results were also obtained in T47D cell line (Figure S5, Supporting Information). These findings suggest that ER*α* can transactivate MIR497HG expression in breast cancer.

### 2.5 MIR497HG Depletion Contributes to Estrogen‐Independent Growth and Tamoxifen Resistance

**Figure 4:**
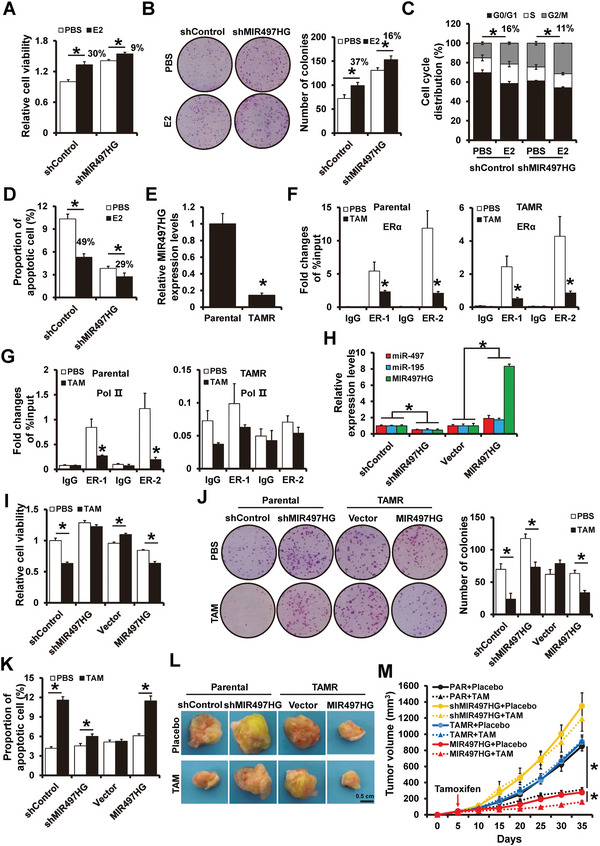
MIR497HG regulates estrogen‐independent growth and tamoxifen resistance in ER+ breast cancer cells. A,B) Proliferation abilities of MIR497HG‐depleted and control cells examined by MTT (A) and colony formation (B) assays. MCF7 cells were cultured in hormone‐deprived conditions for 5 days and subsequently treated with or without 10 nm E2 for 5 days. C) Cell cycle distribution and D) apoptosis of MIR497HG‐depleted and control cells examined via flow cytometry. Hormone‐deprived MCF7 cells were treated with or without 10 nm E2 for 48 h. E) RT‐qPCR results showing levels of MIR497HG mRNA expression in MCF7 parental and TamR cells. F) ChIP‐seq results showing enrichment of ER*α* on the MIR497HG promoter region in MCF7 parental (left) and TamR (right) cells treated with 1 µm tamoxifen for 48 h. G) Enrichment of RNA polymerase II on the MIR497HG promoter region in MCF7 parental (left) and TamR (right) cells treated with 1 µm tamoxifen for 48 h. H) The expression levels of MIR497HG, miR‐497 and miR‐195 in MIR497HG‐depleted MCF cells and MIR497HG‐overexpressed MCF7/TamR cells detected by RT‐qPCR. I,J) Proliferation abilities of MIR497HG‐depleted MCF7 or MIR497HG‐overexpressed TamR and control cells with or without 1 µm tamoxifen treatment were determined via MTT (H) and colony formation (I) assays. K) Apoptosis of MIR497HG‐depleted MCF7, MIR497HG‐overexpressed TamR and control cells with or without 1 µm tamoxifen treatment determined via flow cytometry. L,M) Tumor growth curves of subcutaneous tumor made of MIR497HG‐depleted MCF7 or MIR497HG‐overexpressed TamR and control cells treated with tamoxifen or placebo at the indicated times. The tumors were dissected and photographed at harvest time. *P < 0.05.

Since MIR497HG is regulated by ER*α*, we wanted to see if MIR497HG affects estrogen responsiveness in ER+ breast cancer cells. Stable MIR497HG‐depleted MCF7 cells or shControl cells were incubated in an estrogen‐deprived medium and subsequently treated with estrogen. MIR497HG‐depleted cells exhibited a smaller decrease in cell viability (**Figure** **4**A), a smaller decrease in colony numbers (Figure 4B), and a smaller increase in cell cycle‐arrested cells at the G0/G1 phases (Figure 4C), and a smaller increase in the apoptotic cell population under estrogen‐deprived conditions (Figure 4D) compared to shControl cells. These data demonstrate that MIR497HG depletion results in significant estrogen‐independent growth.

As decreased cellular demand for estrogen is closely related to endocrine resistance in ER+ breast cancer patients,^[^ ^2^, ^27^ ^]^ we investigated whether MIR497HG depletion contributes to tamoxifen resistance using MCF7/TamR (an acquired tamoxifen‐resistant MCF7 cell line) as previously described.^[^ ^28^ ^]^ Compared to the parental control cells, MIR497HG expression was remarkably lower in MCF7/TamR cells (Figure 4E). ChIP analysis suggested that tamoxifen treatment reduced ER*α* binding to the MIR497HG promoter region in either parental MCF7 or MCF7/TamR cells (Figure 4F). Intriguingly, RNA polymerase II occupancy on the MIR497HG promoter region was observed and reduced after tamoxifen treatment in parental MCF7 cells but not in MCF7/TamR cells. These results imply that MIR497HG transcriptional repression occurs in MCF7/TamR cells independent tamoxifen treatment (Figure 4G). To confirm that MIR497HG depletion played a role in tamoxifen resistance, MCF7/TamR cells were used to generate stable MIR497HG‐overexpressed cells and control cells via lentiviral infection (Figure 4H). MIR497HG overexpression caused a greater tamoxifen‐induced reduction in cell viability (Figure 4I), number of colonies (Figure 4J) and increase in the apoptotic cell population (Figure 4K). These data suggest that MIR497HG overexpression improved tamoxifen sensitivity in tamoxifen‐resistant breast cancer cells. MIR497HG depletion, on the other hand, reduced tamoxifen sensitivity in ER+ breast cancer cells (Figure 4I–K). Overexpression of miR‐195/497 remarkably reversed the tamoxifen resistance induced by MIR497HG depletion (Figure S6A–C, Supporting Information). Similarly, miR‐497/195 expression in MCF7/TamR cells was downregulated compared to parental control cells (Figure S7A, Supporting Information). Notably, forced expression of miR‐195/497 increased tamoxifen sensitivity in ER+ breast cancer cells (Figure S7B–E, Supporting Information).

Xenograft experiments were employed to assess the influence of MIR497HG on tamoxifen resistance in vivo. MIR497HG‐depleted MCF7 or MIR497HG‐overexpressed MCF7/TamR, as well as the control cells were injected subcutaneously with exogenous estrogen supplement in female SCID mice. Once the volume tumor achieved ≈100 mm^3^, the mice were given a 30‐day course of slow‐release tamoxifen pellets. Tamoxifen treatment significantly suppressed tumor growth in MCF7‐shControl cells but not in MCF7‐shMIR497HG cells (Figure 4L,M, Supporting Information), demonstrating that MIR497HG depletion reduced tamoxifen sensitivity in ER+ breast cancer cells. Tamoxifen had no effect on the tumor growth derived from MCF7/TamR‐vector cells, but remarkably decreased those derived from MCF7/TamR‐MIR497HG cells (Figure 4L,M, Supporting Information). These results suggest that MIR497HG overexpression reverses tamoxifen resistance in tamoxifen‐resistant breast cancer cells.

### 2.6 MIR497HG Mediates Tamoxifen Sensitivity through the PI3K‐AKT Signaling Pathway

**Figure 5:**
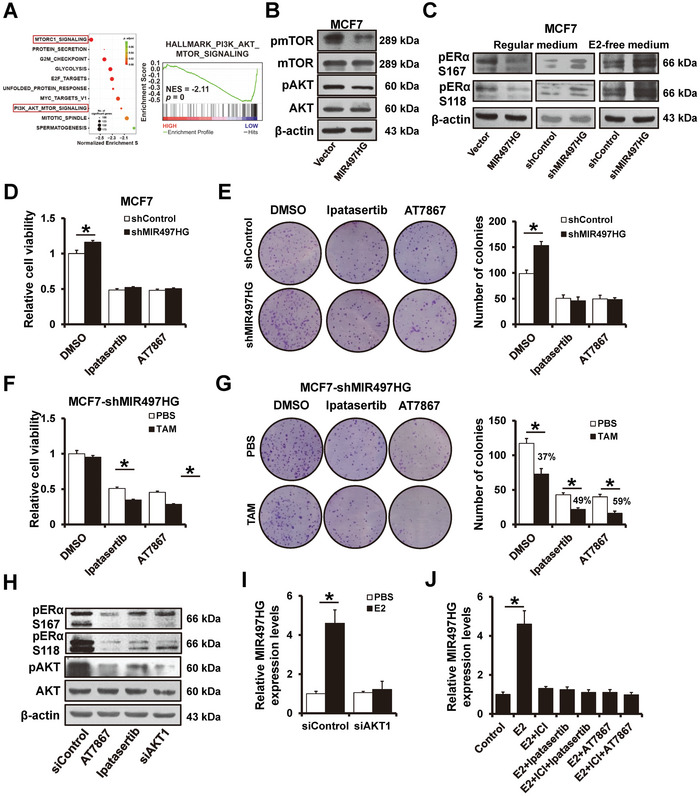
MIR497HG regulates tamoxifen sensitivity via PI3K‐AKT signaling pathway. A) Gene set enrichment analysis results showing the correlation between MIR497HG expression and the PI3K‐AKT signaling gene signatures in a TCAG cohort. B) Western blots showing expression levels of total and phosphorylated AkKT and mTOR proteins in MIR497HG‐overexpressed MCF7 and control cells. C) Western blots showing expression levels of phosphorylated ER *α* protein in MIR497HG‐overexpressed, MIR497HG‐depleted MCF7 and control cells, grown in regular or E2‐deprived media. D,E) Proliferation abilities of MIR497HG‐depleted MCF7 and control cells treated with Ipatasertib or AT7867 determined by MTT (D) and colony formation (E) assays. F,G) Proliferation abilities of MIR497HG‐depleted MCF7 treated with Ipatasertib or AT7867 in the presence of 1 µm tamoxifen were determined by MTT (F) and colony formation (G) assays. H) Western blots showing expression of phosphorylated and total AKT and ER *α* proteins in MCF7 cells treated with Ipatasertib or AT7867 or transfected with siRNAs targeting AKT1. I) RT‐qPCR results showing levels of MIR497HG mRNA expression in MCF7 cells transfected with siRNAs targeting AKT1 in the presence or absence of 10 n m E2. J) RT‐qPCR results showing levels of MIR497HG mRNA expression in indicated MCF7 cells. *P < 0.05.

The Gene Set Enrichment Analysis of the MIR497HG differentially expressed genes revealed significant enrichments in the PI3K‐AKT‐mTOR gene in breast cancer samples with the low MIR497HG expression ( **Figure** **5**A). Tamoxifen resistance is linked to the PI3K‐AKT signaling pathway activation in breast cancer. Thus, we sought to determine whether MIR497HG mediate tamoxifen sensitivity via the PI3K‐AKT signaling. MIR497HG overexpression reduced the phosphorylation of mTOR and AKT in MCF7 cells (Figure 5B). It is well established that AKT can cause tamoxifen resistance through two main pathways, including self‐phosphorylation and ligand‐independent ER*α* activation.^[^ ^29^ ^]^ MIR497HG overexpression decreased ER*α* phosphorylation, whereas MIR497HG depletion increased ER*α* phosphorylation at both the S167 and S118 sites (Figure 5C). Moreover, increased ER*α* phosphorylation was observed in MIR497HG‐depleted MCF7 cells in E2‐free medium (Figure 5C), indicating that MIR497HG knockdown causes ligand‐independent ER*α* phosphorylation. MIR497HG depletion did not influence the cell viability (Figure 5D) or colony numbers (Figure 5E) in MCF7 cells after treatment with AKT signaling pathway inhibitors (Ipatasertib and AT7867). These results indicate that MIR497HG functions in an AKT signaling‐dependent manner. Furthermore, inhibiting the AKT signaling pathway increased tamoxifen sensitivity of MIR497HG‐depleted MCF7 cells (Figure 5F,G) while decreasing ER*α* phosphorylation (Figure 5H). Because ER*α* phosphorylation is required for ER*α* activation, we studied the effect of the AKT signaling pathway on ER*α*‐mediated MIR497HG expression. Inhibiting the AKT signaling pathway with siRNAs or inhibitors significantly reduced E2‐induced MIR497HG expression in MCF7 cells (Figure 5I,J). However, AKT inhibition did not influence MIR497HG expression after ER*α* degradation with fulvestrant (Figure 5J). These data suggest that MIR497HG regulation via the AKT signaling pathway is E2/ER*α* dependent.

Next, we looked into the function of the AKT signaling pathway on tamoxifen resistance in MCF7/TamR cells. Overexpression of miR‐497 or miR‐195 reduced the phosphorylation of mTOR, AKT, and ER *α* in MCF7/TamR cells (Figure S8A, Supporting Information). Forced expression of miR‐195/497 did not affect cell viability (Figure S8B, Supporting Information) and colony numbers (Figure S8C, Supporting Information) in MCF7/TamR cells following treatment with AKT signaling pathway inhibitors. Furthermore, siRNAs or inhibitors of the AKT signaling pathway significantly reduced E2‐induced miR‐497 (Figure S8D,F, Supporting Information) and miR‐195 (Figure S8E,F, Supporting Information) expression in MCF7/TamR cells. AKT inhibition did not influence miR‐497 or miR‐195 expression during fulvestrant‐induced ER*α* degradation (Figure S8F, Supporting Information).

### 2.7 Identification of miR‐497/195 Cluster Targets

**Figure 6:**
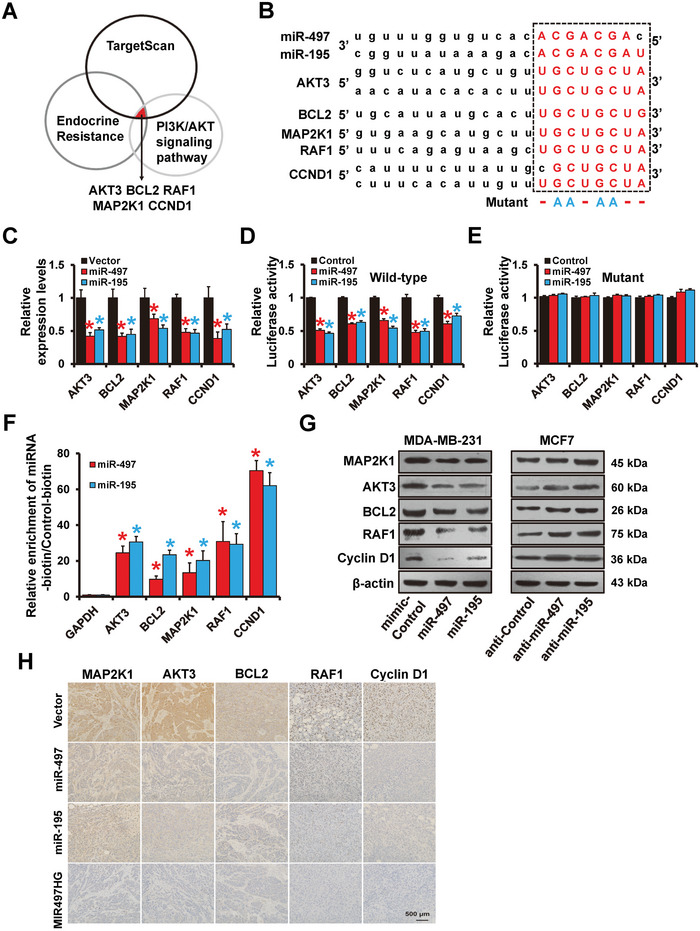
Identification of miR‐497/195 targets in breast cancer. A) Potential miR‐497/195 targets detected via TargetScan and KEGG analyses. B) The predicted binding of miR‐497/195 with the AKT3, BCL2, MAP2K1, RAF1 or CCND1 3′‐UTR. C) RT‐qPCR results showing levels of AKT3, BCL2, MAP2K1, RAF1, and CCND1 mRNA expression in indicated MDA‐MB‐231 cells. D,E) Results of the dual‐luciferase reporter assay verifying AKT3, BCL2, MAP2K1, RAF1, and CCND1 as the miR‐497/195 targets. A 3′‐UTR region containing the predicted wild‐type (D) miR‐497/195 target sites or the corresponding mutated reporter plasmids (E) were constructed and then transfected into 293FT cells with or without miR‐497 or miR‐195 mimics. F) RT‐qPCR results showing enrichment of miR‐497/195 targets in MCF7 cells transfected with biotin‐tagged miR‐497, miR‐195, and control RNA. G) Western blots showing expression levels of AKT3, BCL2, MAP2K1, RAF1, and CCND1 proteins in miR‐497/195‐overexpressed MDA‐MB‐231, miR‐497/195‐depleted MCF7, and control cells. H) Immunohistochemical results showing levels of AKT3, BCL2, MAP2K1, RAF1, and CCND1 expression in indicated tumors from mice. *P < 0.05.

Bioinformatics analysis with TargetScan was performed to predict mRNA targets to identify downstream targets of the miR‐497/195 cluster. When the predicted targets were compared to the set of endocrine resistance pathway genes or the PI3K‐AKT signaling pathway genes (**Figure** **6**A), five putative target genes were identified, including *AKT3*, *BCL2*, *RAF1*, *MAP2K1*, and *CCND1* (Figure 6B). Consistent with bioinformatics prediction, forced expression of miR‐195/497 reduced mRNA levels of *AKT3*, *BCL2*, *RAF1*, *MAP2K1*, and *CCND1* expression in MDA‐MB‐231 cells (Figure 6C). Luciferase reporter assays illustrated that miR‐497/195 overexpression reduced the luciferase activities of each of the five genes (Figure 6D). Mutations were introduced into the miR‐195/497 binding sites of the luciferase reporter plasmids (Figure 6B) and mutation of the predicted binding sites abrogated the miR‐195/497‐induced decrease in luciferase reporter activities (Figure 6E). Furthermore, enrichment of these five genes was observed when mRNAs were pulled down with biotinylated miR‐497/195 mimics (Figure 6F), demonstrating that miR‐195/497 directly bind to these sites. Also, forced expression of miR‐195/497 decreased, whereas miR‐195/497 depletion upregulated these five target genes in MCF7 cells and MDA‐MB‐231, respectively (Figure 6G). Immunohistochemical staining revealed that the expression of *AKT3*, *BCL2*, *RAF1*, *MAP2K1*, and *CCND1* was lower in tumors from 231‐MIR497HG, 231‐miR‐497, or 231‐miR‐195 cells than in tumors from 231‐control mice (Figure 6H). These findings demonstrate that *AKT3*, *BCL2*, *RAF1*, *MAP2K1*, and *CCND1* are miR‐497/195 cluster targets.

### 2.8 ZEB1‐Mediated Promoter Hypermethylation Represses MIR497HG Transcriptional Activity

**Figure 7:**
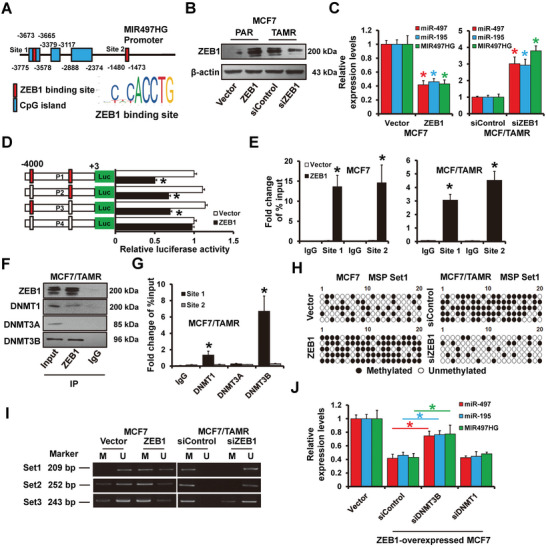
ZEB1‐mediated DNA methylation on MIR497HG promoter. A) Schematic representation of the MIR497HG promoter region. B) Western blots showing expression levels of ZEB1 protein in MCF7/parental cells transfected with ZEB1 expressing plasmid, or MCF7/TamR cells transfected with siRNAs targeting ZEB1 and control cells. C) Levels of MIR497HG and miR‐497/195 expression in cells as in (B) determined by RT‐qPCR. D) Dual‐luciferase reporter assay results indicated that ZEB1 regulates MIR497HG promoter activity. E) ChIP‐seq analysis results showing enrichment of ZEB1 on the MIR497HG promoter region in MCF7/parental (left) transfected with ZEB1 expressing plasmid and MCF7/TamR (right) cells. Enrichment of MIR497HG promoter DNA in the ZEB1‐immunoprecipitated samples determined by qPCR. F) The relationship between ZEB1 with endogenous DNMTs in MCF7/TamR cells. The immunoprecipitants were subjected to western blot analysis targeting an anti‐DNMT1, DNMT3A, and DNMT3B antibodies. G) ChIP‐seq analysis results illustrating enrichment of DNMTs on the MIR497HG promoter region in MCF7/TamR cells. qPCR results showing enrichment of MIR497HG promoter DNA in the DNMTs‐immunoprecipitated samples. H) Bisulfite sequence analysis of MCF7/parental transfected with ZEB1 expressing plasmid (left) or MCF7/TamR cells transfected with siRNAs targeting ZEB1 (right), and control cells. I) Methylation specific PCR of bisulfite‐modified DNA in cells as in (H). J) RT‐qPCR results showing expression levels of MIR497HG and miR‐497/195 in ZEB1‐overexpressed MCF7 transfected with siRNAs targeting DNMT3B or DNMT1. *P < 0.05.

Previous research has shown that ZEB1 can bind to the miR‐190 promoter and cause breast cancer tamoxifen resistance.^[^ ^30^ ^]^ In this view, we sought to know if ZEB1 regulates MIR497HG. The analysis revealed two canonical E‐boxes (CACCTG) on the MIR497HG region (**Figure** **7**A). ZEB1 expression was upregulated in MCF7/TamR cells (Figure 7B). Furthermore, ZEB1 overexpression decreased the miR‐497/195 and MIR497HG expression in MCF7 cells, whereas ZEB1 knockdown increased their expression in MCF7/TamR cells compared to control cells (Figure 7B,C). Next, we performed a luciferase reporter assay to see how ZEB1 expression influenced MIR497HG promoter activity. As shown in Figure 7D, ZEB1 overexpression decreased the wild‐type (P1) MIR497HG promoter activity, but this effect was abolished in the mutated MIR497HG promoter, where the ZEB1 binding sites were inactivated (P4) by site‐directed mutagenesis. ChIP analysis showed that ZEB1 could bind to the MIR497HG promoter (Site 1 and Site 2) in ZEB1‐overexpressed MCF7 cells (Figure 7E; left) and MCF7/TamR cells (Figure 7E; right). After recruiting DNMTs to the MIR497HG promoter region, we investigated whether ZEB1 regulation of MIR497HG was dependent on DNMTs. As illustrated in Figure 7F, DNMT1 and DNMT3B were co‐immunoprecipitated with ZEB1 in MCF7/TamR cells. ChIP analysis demonstrated that DNMT1 and DNMT3B could bind to site 1 on the MIR497HG promoter region but not site 2 (Figure 7G). DNA methylation was higher in ZEB1‐overexpressed MCF7 cells (Figure 7H; left). By BSP analysis, ZEB1 depletion in MCF7/TamR cells resulted in decreased DNA methylation (Figure 7H; right). According to methylation‐specific PCR, ZEB1 overexpression in MCF7 cells increased DNA methylation of the MIR497HG promoter, whereas ZEB1 depletion in MCF7/TamR cells decreased this methylation (Figure 7I). Furthermore, DNMT3B depletion could abolish the ZEB1‐induced repression of miR‐497/195 and MIR497HG expression in MCF7 cells (Figure 7J). These observations revealed that ZEB1 represses the expression of miR‐497/195 and MIR497HG by recruiting DNMT3B to the MIR497HG promoter.

### 2.9 ZEB1 Recruits HDAC1/2 on the MIR497HG Promoter and Represses Its Activity

**Figure 8:**
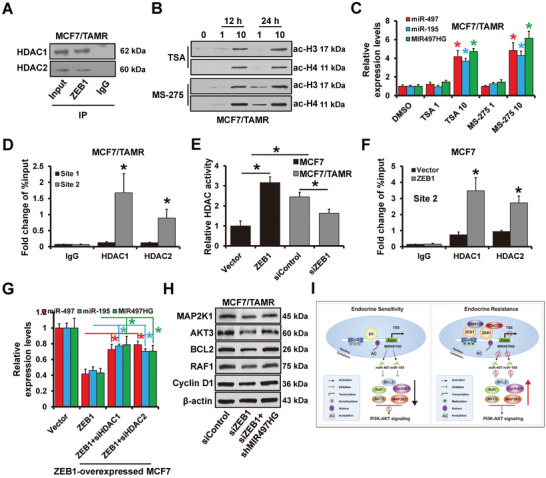
ZEB1 Recruits HDAC1/2 to MIR497HG promoter. A) Relationship between ZEB1 with endogenous HDAC1/2 in MCF7/TamR cells. The cells were subjected to co‐immunoprecipitation using an anti‐ZEB1 or a normal control IgG antibody, and the immunoprecipitants analyzed via western blot assay targeting an anti‐HDAC1 and HDAC2 antibody. B) Western blots showing expression levels of acetylated histones H3 and H4 in MCF7/TamR cells treated with TSA or MS‐275. C) RT‐qPCR indicating expression levels of MIR497HG and miR‐497/195 in MCF7/TamR cells treated with TSA or MS‐275. D) ChIP‐seq analysis results showing enrichment of HDAC1/2 on the MIR497HG promoter region in MCF7/TamR cells. E) HDAC activity in MCF7/parental cells transfected with ZEB1 expressing plasmid, MCF7/TamR cells transfected with siRNAs targeting ZEB1 and control cells. F) ChIP‐seq analysis results showing enrichment of HDAC1/2 on the MIR497HG promoter region in ZEB1‐overexpressed MCF7 and control cells. G) RT‐qPCR results indicating expression levels of MIR497HG and miR‐497/195 in ZEB1‐overexpressed MCF7 cells transfected with siRNAs targeting HDAC1 or HDAC2. H) The expression levels of MAP2K1, AKT3, BCL2, RAF1, and CCND1 in ZEB1‐depleted and MIR497HG‐depleted MC7/TamR cells detected by western blot. I) A model of MIR497HG in ER+ breast cancer tamoxifen sensitivity. *P < 0.05.

As previously described, ZEB1 recruits DNMT3B on site 1 (but not site 2) of the MIR497HG promoter region. However, neither site 1‐ nor site 2 mutations abolished ZEB1 repression on the MIR497HG promoter (Figure 7D), implying that ZEB1 regulates MIR497HG transcriptional activity via another mechanism. Because ZEB1 recruits and represses HDAC1/2 expression on the E‐cadherin promoter,^[^ ^31^ ^]^ we investigate whether it can also recruit HDAC1/2 on the MIR497HG promoter. The immunoprecipitation (IP) analysis demonstrated that HDAC1 and HDAC2 co‐immunoprecipitated with ZEB1 in MCF7/TamR cells (**Figure** **8**A). We revealed elevated acetylation of histones H3/H4 in MCF7/TamR cells treating with the HDAC inhibitors MS‐275 or TSA for 12–24 h (Figure 8B). Furthermore, after 48 h of incubation with TSA or MS‐275, the expression of miR‐195/497 and MIR497HG was upregulated in MCF7/TamR cells (Figure 8C). ChIP analysis demonstrated that HDAC1/2 could bind to site 2, but not site 1 of the MIR497HG promoter region (Figure 8D). Furthermore, compared to control group, the total HDAC enzymatic activity was remarkably elevated in ZEB1‐overexpressed MCF7 cells, whereas it was decreased in ZEB1‐depleted MCF7/TamR cells (Figure 8E). ZEB1 overexpression increased HDAC1/2 occupancy on site 2 of the MIR497HG promoter in MCF7 cells (Figure 8F). Furthermore, HDAC1/2 depletion could reverse ZEB1‐induced repression of miR‐497/195 and MIR497HG expression in MCF7 cells (Figure 8G). Besides, the decrease of PI3K‐AKT signaling regulators caused by siZEB1 could be reversed by MIR497HG depletion (Figure 8H), indicating ZEB1 activated PI3K‐AKT signaling by regulating MIR497HG expression. These findings suggest that ZEB1 represses the expression of miR‐497/195, and MIR497HG by recruiting HDAC1/2 to the MIR497HG promoter, thus, regulating PI3K‐AKT signaling.

## 3 Discussion

One‐third of patients with breast cancer relapse due to endocrine resistance despite receiving antiestrogen therapy, and this remains one of the major obstacles to effective therapy in ER+ breast cancer.^[^ ^32^ ^]^ Consequently, a further investigation of the mechanisms of antiestrogen resistance, particularly tamoxifen resistance, is urgently needed. Herein, we discovered a novel model in which MIR497HG depletion activates PI3K‐AKT signaling, causing tamoxifen resistance (Figure 8I).

MIR497HG functions as a polycistronic miRNA host gene with the first intron encoding miR‐497 and miR‐195. MIR497HG was first reported to dysregulation in bladder cancer and identified to be an emerging diagnostic marker.^[^ ^14^ ^]^ MIR497HG deficiency promotes cancer progression by regulating transforming growth factor‐beta/Smad and Hippo/Yap signaling in bladder cancer.^[^ ^13^ ^]^ Decreased expression of miR‐497/195 is involved in cancer progression in clinical specimens, and they act as tumor suppressors in breast cancer, as well as several other kinds of human malignances.^[^ ^25^ ^]^ The present research revealed that MIR497HG expression is decreased in breast cancer tissues, and patients expressing high MIR497HG levels had a better prognosis than those expressing low MIR497HG levels. MIR497HG expression was positively correlated with miR‐195/497 expression and MIR497HG depletion significantly downregulated miR‐195/497 expression in breast cancer cells, supporting previous research that MIR497HG is a precursor of miR‐195 and miR‐497.^[^ ^13^ ^]^ Moreover, miR‐195/497 abrogated the MIR497HG depletion‐induced promotion of cell proliferation, invasion, migration, and EMT‐like phenotype.

Recent evidence has demonstrated a function for lncRNAs in breast cancer tamoxifen resistance, but the mechanism by which lncRNAs regulate tamoxifen resistance remains unclear.^[^ ^33^, ^34^, ^35^ ^]^ Here, we demonstrated that MIR497HG expression was significantly reduced in tamoxifen‐resistant breast cancer cell lines and that MIR497HG overexpression sensitized tamoxifen‐resistant cells to tamoxifen treatment. In addition, overexpression of miR‐497 or miR‐195 increased tamoxifen sensitivity in tamoxifen‐resistant cells, implying that MIR497HG reversed tamoxifen resistance in breast cancer through upregulation of miR‐195/497. Aberrant activation of the PI3K‐AKT signaling pathway resulted in acquired endocrine resistance. Evidence shows that the PI3K‐AKT signaling pathway is an emerging target for many new therapeutic agents for patients with ER+ breast cancer.^[^ ^29^, ^36^ ^]^ Here, we found a novel regulatory mechanism involving MIR497HG and ER*α*/PI3K‐AKT interactions. Reduced MIR497HG expression resulted in increased levels of miR‐497/195 target genes (AKT3, BCL2, MAP2K1, RAF1, and CCND1), which activated the of PI3K‐AKT signaling and, as a result, increased ER*α* transcriptional activity, inducing tamoxifen resistance.^[^ ^37^ ^]^ Inhibiting the AKT signaling pathway decreased ER*α* transcriptional activity to sensitize MIR497HG‐depleted breast cancer cells to tamoxifen treatment. Therefore, MIR497HG was identified as a critical regulator of the signaling cross‐talk between ER*α* and PI3K‐AKT in ER+ breast cancer.

Increasing evidence shows that epigenetic modifications, including histone acetylation and DNA methylation, perform crucial functions in tamoxifen sensitivity in breast cancer.^[^ ^3^ ^]^ DNMTs have been identified as a key regulator that could affect human genome DNA methylation, which has been identified as an emerging prognostic factor for predicting tamoxifen response in patients with breast cancer.^[^ ^38^, ^39^ ^]^ DNMT3B expression was reported to be an independent prognostic biomarker for survival in tamoxifen‐treated breast cancer patients.^[^ ^40^ ^]^ HDACs form protein complexes with other proteins and act as transcription repressors. HDACs with abnormal expression or activity are observed in many human malignances, including breast cancer. Several types of HDAC inhibitors have been exploited to suppress the effect of HDAC on breast cancer antiestrogen resistance.^[^ ^41^, ^42^ ^]^ Here, we found that DNA hypermethylation and histone deacetylation cause MIR497HG repression. So far, the DNA hypomethylating agent 5‐aza‐2′‐deoxycytodine (decitabine) has been applied in clinical trials, and histone deacetylase inhibitor (HDACI) is also a hot spot in clinical development. However, the application of AZA or HDACI in breast cancer endocrine therapy remains to be clarified. AZA or HDAC1/2 inhibitors could induce MIR497HG expression in MCF7/TamR cells. ZEB1, a master EMT inducer, is upregulated in breast cancer, contributing to metastasis and drug resistance.^[^ ^43^, ^44^ ^]^ ZEB1 confers resistance to endocrine therapy by inducing ER*α* promoter hypermethylation.^[^ ^45^ ^]^ We also demonstrated that ZEB1 interplayed with HDAC1/2 and DNMT3B at the MIR497HG promoter, resulting in promoter hypermethylation and histone deacetylation, which can be reversed by ZEB1 depletion. Thus, our data demonstrate that ZEB1 can regulate MIR497HG expression by recruiting of DNMT3B and HDAC1/2 to the MIR497HG promoter, resulting in transcriptional repression. Interestingly, in our study, MIR497HG can be regulated by both ER*α* transactivation and ZEB1 epigenetic inhibition, which perform dynamic roles in endocrine sensitivity of breast cancer. It is suspected that in endocrine sensitive status, MIR497HG is mainly activated by ER*α* and results in the downregulation of miR‐497/195, thus inhibits PI3K‐AKT signaling. While in endocrine resistant status, DNMT3B and HDAC1/2 recruited by ZEB1 are combined to the promoter region of MIR497HG and lead to its downregulation, the overexpression of miR‐497/195 activates PI3K‐AKT signaling and induces endocrine resistance (Figure 8I).

In conclusion, this work presents a novel mechanism underlying tamoxifen resistance caused by ZEB1‐mediated histone deacetylation and DNA methylation of the MIR497HG promoter. miR‐497 and miR‐195 work together to inhibit PI3K‐AKT signaling by downregulating the five positive regulators of PI3K‐AKT signaling. We propose that MIR497HG can serve as a prognostic factor for tamoxifen sensitivity in patients with ER+ breast cancer. Nevertheless, a large sample of clinical data is required to determine the prognostic value of MIR497HG in clinical application.

## 4 Experimental Section

### Oligonucleotides, Plasmid, and Transfection

The miR‐195 or miR‐497 mimics/inhibitor, shMIR497HG, siRNAs targeting ER*α*, ZEB1, AKT1, DNMT1, DNMT3B, HDAC1, HDAC2, and corresponding controls were purchased from RiboBio (China), the oligonucleotides of which are listed in Table S1 of the Supporting Information, including the MIR497HG promoter regions (−4000 to +83, −1000 to +83, −457 to +83, and −333 to +83) and the ER*α* or ZEB1 binding site‐mutated promoter region. These sequences were inserted into the pGL3‐basic vector (Promega, Madison, WI). The sequences that contain predicted miRNA binding sites or corresponding mutants were synthesized and inserted into the psiCHEK2 vector (Promega) for miRNA target gene luciferase reporter. The mutant constructs were created using a site‐directed mutagenesis kit (Transgen, China). Furthermore, human MIR497HG and ZEB1 cDNAs were synthesized, and they were synthesized into the pcDNA3 vector. Transient transfections were conducted following the standard protocol provided by FuGENE HD Transfection Reagent (Promega), with each transfection system containing 2 µg plasmid DNA. Stable transfections were conducted with specific lentiviruses (RiboBio), followed by puromycin (2 µg mL^−1^) selection of infected cells for at least one week.

### Cell Culture and Reagents

Seven breast cancer cell lines (BT474, BT549, MCF7, SKBR3, MDA‐MB‐231, MDA‐MB‐468, and T47D), one normal breast cell line (MCF10A) and 293FT were purchased from the Cell Bank of the Chinese Academy of Sciences (China). MCF7 with tamoxifen resistance, dubbed MCF7/TamR was developed by treating MCF7 cells with tamoxifen (0.1 µm) for 6 months. MCF7/TamR cells were cultured with 0.1 µm tamoxifen to maintain tamoxifen resistance. All cells were cultured as previously described.^[^ ^28^, ^46^ ^]^ Tamoxifen, fulvestrant (ICI‐182780), 5′‐AZA, Trichostatin A (TSA), MS275, Ipatasertib, and AT7867 were purchased from Selleck, while estradiol was from Sigma‐Aldrich (St. Louis, MO). The hormone‐deprived serum was prepared with dextran‐coated charcoal (Sigma‐Aldrich). Cells were cultured in phenol red‐free medium (Life Technologies, Grand Island, NY) with 5% hormone‐deprived serum for 5 days prior to estrogen stimulation and tamoxifen treatment to reveal estrogen‐related signaling pathways.

### Reverse Transcription‐Quantitative Polymerase Chain Reaction

mRNA and miRNA were extracted using TRIzol Reagent (Life Technologies) or mirVana miRNA Isolation kit (Life Technologies) according to the protocols. The expression levels of mRNA or miRNA were determined by GoTaq qPCR Master Mix (Promega) or TaqMan miRNA assay kit (Life Technologies) as previously described.^[^ ^47^ ^]^ GADPH expression was employed to normalize the target gene expression. Table S2 of the Supporting Information outlines the primers sequences.

### Western Blot and Immunohistochemistry Assays

Cells were lysed in ice‐cold RIPA buffer after proposed experiments and proteins were resolved by SDS‐PAGE and then transferred to a PVDF membrane, which was incubated with primary antibodies at 1:1000 dilution. HRP‐conjugated secondary antibody incubation at a dilution of 1:5000 was applied after three washes with 1× TBST buffer for 60 min. ECL reagent (Millipore, Bedford, MA) was used to visualize protein bands.

For immunohistochemistry analysis, formalin‐fixed, paraffin‐embedded sections were deparaffinized, rehydrated, and antigen retrieved by boiling in sodium citrate buffer. The sections were incubated overnight at 4 °C with primary antibodies at 1:100 dilution, and then exposed to HRP‐conjugated secondary antibody at 1:500 dilution and covered with 3,3′‐diaminobenzidine. The slides were examined using a light microscope and images were captured using a microscopy camera. Table S3 of the Supporting Information shows the antibodies to the examined proteins that were studied.

### Flow Cytometry Analysis and Cell Function Assays

Flow cytometry analysis was conducted to explore cell cycle distribution and cell apoptosis as previously described.^[^ ^48^ ^]^ Colony formation and MTT assays were performed to assess cell proliferation ability. Cell invasion and migration were assessed using transwell and wound healing assays. All experiments were performed as previously described.^[^ ^47^, ^49^ ^]^

### Biotinylated miRNA Pull‐Down and Dual‐Luciferase Reporter

Biotinylated miR‐195 or miR‐497 mimics were used for transfection of MDA‐MB‐231 cells. Then, cell lysates were incubated for 3 h with magnetic beads (Life Technologies) at 4 °C and then washed in wash buffer. Next, 50 µL of the cell lysates was aliquoted for input. The bound RNAs were purified using the RNeasy Mini kit (QIAGEN) and tested using RT‐qPCR with primers targeting miR‐497/195‐binding sites. Dual‐luciferase reporter assay was conducted as previously described.^[^ ^28^ ^]^

### IP and Chromatin Immunoprecipitation‐qPCR

For immunoprecipitation analysis, RIPA buffer was used to lyse cells. Then, the cell lysate supernatant was incubated overnight with the proposed primary antibody at 4 °C. Protein A/G agarose beads (Santa Cruz, Santa Cruz, CA) were used to collect precipitated proteins, which were then washed and resuspended in a pre‐prepared IP buffer containing EDTA (0.1 mm), NaCl (50 mm), and Tris‐HCl (50 mm). Protein samples were then western blotted using protocols described above.

ChIP‐qPCR analysis was conducted in accordance with the protocols (Millipore), using anti‐ER*α*, anti‐RNA polymerase II, anti‐DNMT1, anti‐HDAC1, anti‐DNMT3A, anti‐HDAC2, anti‐DNMT3B, or an isotype control.^[^ ^28^ ^]^ Precipitated DNA was processed by qPCR using corresponding primers (Table S4, Supporting Information). The data were presented as a percentage of the input.

### Bisulfite Sequencing and Methylation‐Specific PCR

DNA from breast cancer cells was extracted using the Genomic DNA Extraction Kit (Thermo, Waltham, MA), which was then converted by the DNA Methylation‐Gold Kit (Zymo Research, Irvine, CA). The PCR products were resolved on a 2% agarose gel with ethidium bromide, and the purified PCR fragments were cloned into a pGEM‐T easy vector (Promega). Separated clones were thereafter sequenced. Bisulfite‐converted DNA was amplified with specific primers (Table S4, Supporting Information).

### Xenograft

Cells (1 × 10^7^) were subcutaneously inoculated in female NOD/SCID/IL2 receptor *γ* null (NSG) mice (5–7 weeks years old). After the tumor volume achieved 90–110 mm^3^, the mice were divided into 2 groups randomly and, subcutaneously implanted with placebo or tamoxifen pellets for an additional 4 weeks. Each group (6 mice per group), for a total number of 96 mice were involved in the experiment process. The tumor size was examined every 5 days using electron vernier calipers, and the tumor volume was computed using the formula [*V* = (*L* × *W*)^2^/2]. After 35 days, the animals were terminated, and the weight and size of tumor tissue were measured. Breast cancer cells (5 × 10^5^) were intravenously injected into NSG mice. Metastatic clone formation was assessed by bioluminescence imaging every 7 days via in vivo imaging systems. All animal experiments are approved by the Animal Ethics Committee and met the animal welfare guidelines.

### Statistical Analysis

SPSS 24.0 (IBM, Armonk, US) was employed for data analysis. All measurement data were exhibited as mean ± standard deviation. Statistically significant deficiencies between groups were determined by one‐factor analysis of variance (ANOVA) or Student's *t*‐test. A double‐tailed *p*‐value of less than 0.05 denoted statistical significance.

### Ethical Statement

This study was approved by the Institutional Review Board of Tianjin Medical University Cancer Institute and Hospital. Voluntary written informed consent was obtained from each participant prior to inclusion in the study.

## Conflict of Interest

The authors declare no conflict of interest.

## Author Contributions

Y.T., Z.‐H.C., and P.W. contributed equally to this work. X.‐C.C. and Y.Y. designed the study. Y.T., Z.‐H.C., P.W., D.Z., Y.M., X.‐F.L., and X.W. performed the experiments and statistical analysis. Y.T., X.‐C.C., and Y.Y. wrote and revised the manuscript. All authors read and approved the final manuscript.

## Supporting information

Supporting Information

## Data Availability

The data that support the findings of this study are available from the corresponding author upon reasonable request.
