# Supplementary material for: MIR497HG‐Derived miR‐195 and miR‐497 Mediate Tamoxifen Resistance via PI3K/AKT Signaling in Breast Cancer
Source: Adv Sci (Weinh). 2023 Feb 23;10(12):2204819. doi: 10.1002/advs.202204819 (PMC10131819; doi:10.1002/advs.202204819)
Supplement: Supplementary file 1 — Supporting Information [file ADVS-10-2204819-s001.pdf]

ADVANCED SCIENCE
Open Access

Supporting Information

for *Adv. Sci.*, DOI 10.1002/advs.202204819

MIR497HG-Derived miR-195 and miR-497 Mediate Tamoxifen Resistance via PI3K/AKT Signaling in Breast Cancer

Yao Tian, Zhao-Hui Chen, Peng Wu, Di Zhang, Yue Ma, Xiao-Feng Liu, Xin Wang, Dan Ding, Xu-Chen Cao and Yue Yu**

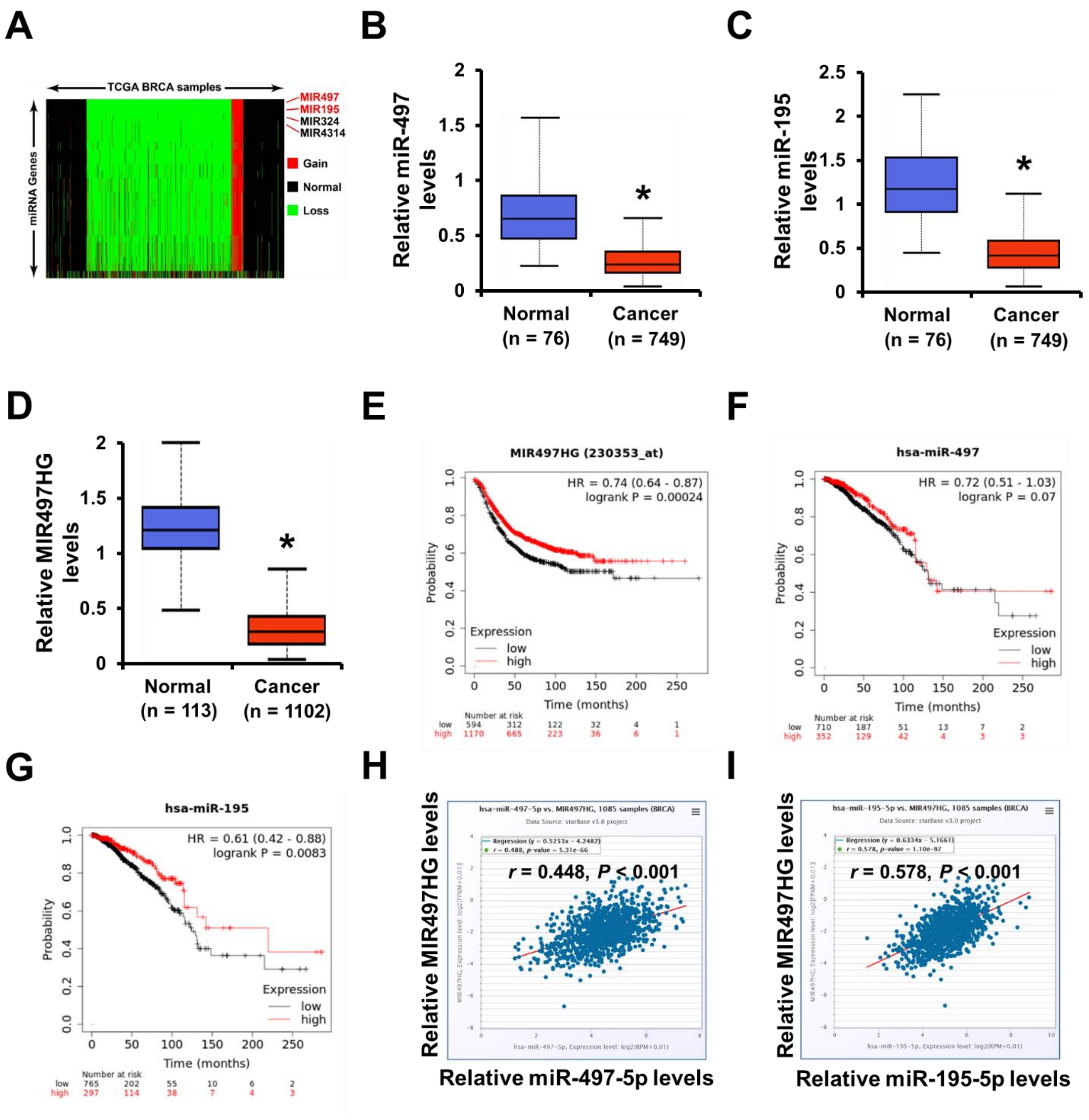

Figure S1. Depletion of MIR497HG or miR-497/195 is correlated with poor prognosis in breast cancer. A, Heat map representing the copy number changes of MIR497HG or miR-497/195 on human chromosome 17p in the TCGA breast cancer. **B-D,** The miR-497 (B), miR-195 (C) and MIR497HG (D) expression in TCGA breast invasive carcinoma and normal tissues. **E-G,** The association between MIR497HG (E)/miR-497 (F)/miR-195 (G) expression and overall survival of patients with breast cancer analyzed by KM-plotter. **H and I,** The relationship between MIR497HG and miR-497 (H)/miR-195 (I) expression in TCGA breast invasive carcinoma. *$P < 0.05$.

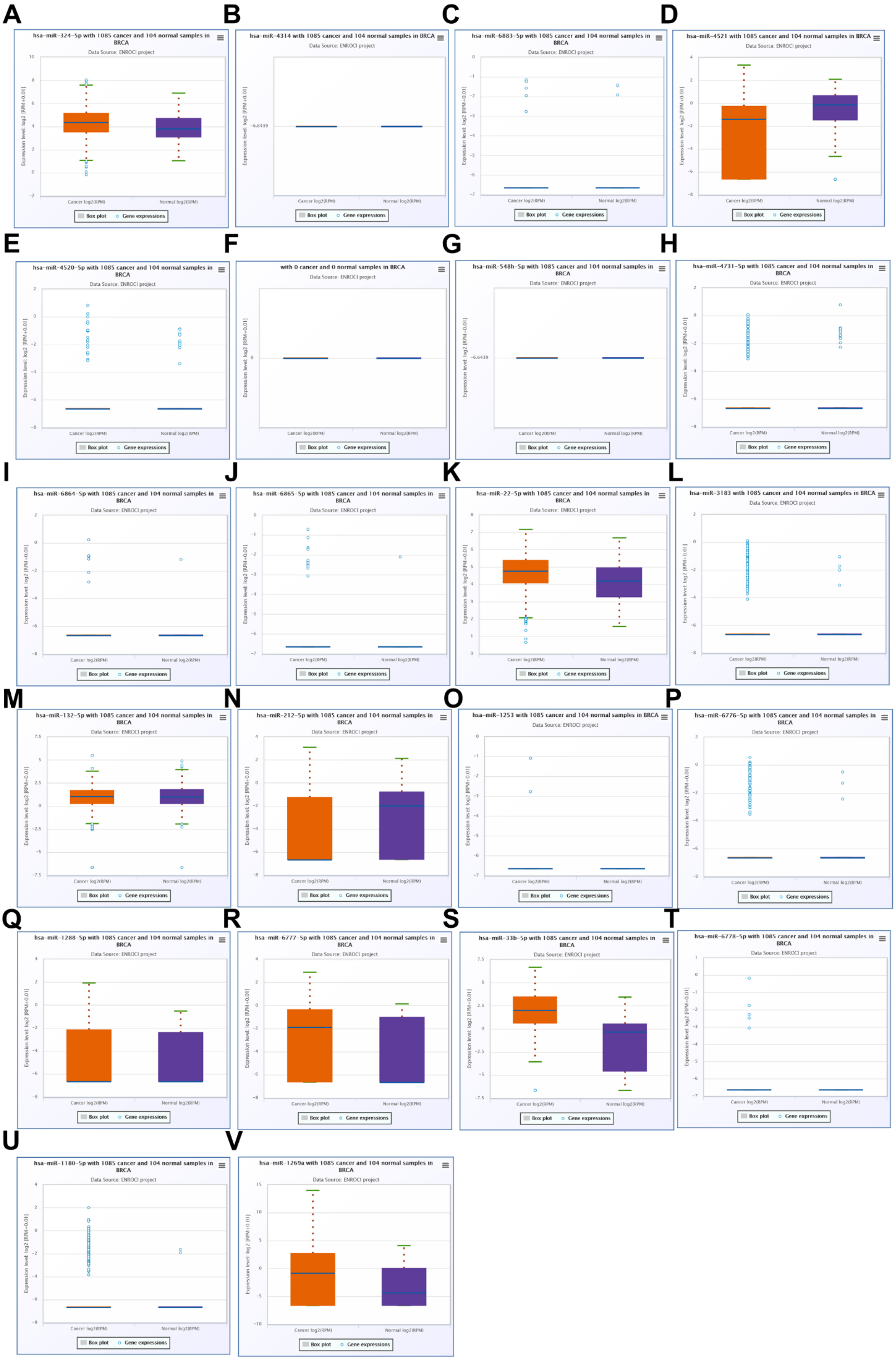
A
hsa-miR-324-5p with 1085 cancer and 104 normal samples in BRCA
B
hsa-miR-4314 with 1085 cancer and 104 normal samples in BRCA
C
hsa-miR-6883-5p with 1085 cancer and 104 normal samples in BRCA
D
hsa-miR-4521 with 1085 cancer and 104 normal samples in BRCA
E
hsa-miR-4520-5p with 1085 cancer and 104 normal samples in BRCA
F
with 0 cancer and 0 normal samples in BRCA
G
hsa-miR-548h-5p with 1085 cancer and 104 normal samples in BRCA
H
hsa-miR-4731-5p with 1085 cancer and 104 normal samples in BRCA
I
hsa-miR-6864-5p with 1085 cancer and 104 normal samples in BRCA
J
hsa-miR-6865-5p with 1085 cancer and 104 normal samples in BRCA
K
hsa-miR-22-5p with 1085 cancer and 104 normal samples in BRCA
L
hsa-miR-3183 with 1085 cancer and 104 normal samples in BRCA
M
hsa-miR-132-5p with 1085 cancer and 104 normal samples in BRCA
N
hsa-miR-212-5p with 1085 cancer and 104 normal samples in BRCA
O
hsa-miR-1253 with 1085 cancer and 104 normal samples in BRCA
P
hsa-miR-6776-5p with 1085 cancer and 104 normal samples in BRCA
Q
hsa-miR-1288-5p with 1085 cancer and 104 normal samples in BRCA
R
hsa-miR-6777-5p with 1085 cancer and 104 normal samples in BRCA
S
hsa-miR-33b-5p with 1085 cancer and 104 normal samples in BRCA
T
hsa-miR-6778-5p with 1085 cancer and 104 normal samples in BRCA
U
hsa-miR-1180-5p with 1085 cancer and 104 normal samples in BRCA
V
hsa-miR-1269a with 1085 cancer and 104 normal samples in BRCA
Data Source: ENROCI project
Cancer log2(RPM)
Normal log2(RPM)
Box plot
Gene expressions

Figure S2. The expression levels of miRNAs on chromosome 17p in breast cancer. The expression levels of **A,** miR-325-5p, **B,** miR-4314, **C,** miR-6883-5p, **D,** miR-4521, **E,** miR-4520-5p, **F,** miR-4520B (no data), **G,** miR-548h-5p, **H,** miR-4371-5p, **I,** miR-6864-5p, **J,** miR-6865-5p, **K,** miR-22-5p, **L,** miR-3183, **M,** miR-132-5p, **N,** miR-212-5p, **O,** miR-1253, **P,** miR-6776-5p, **Q,** miR-1288-5p, **R,** miR-6777-5p, **S,** miR-33b-5p, **T,** miR-6778-5p, **U,** miR-1180-5p and **V,** miR-1269a based on the TCGA dataset in breast cancer.

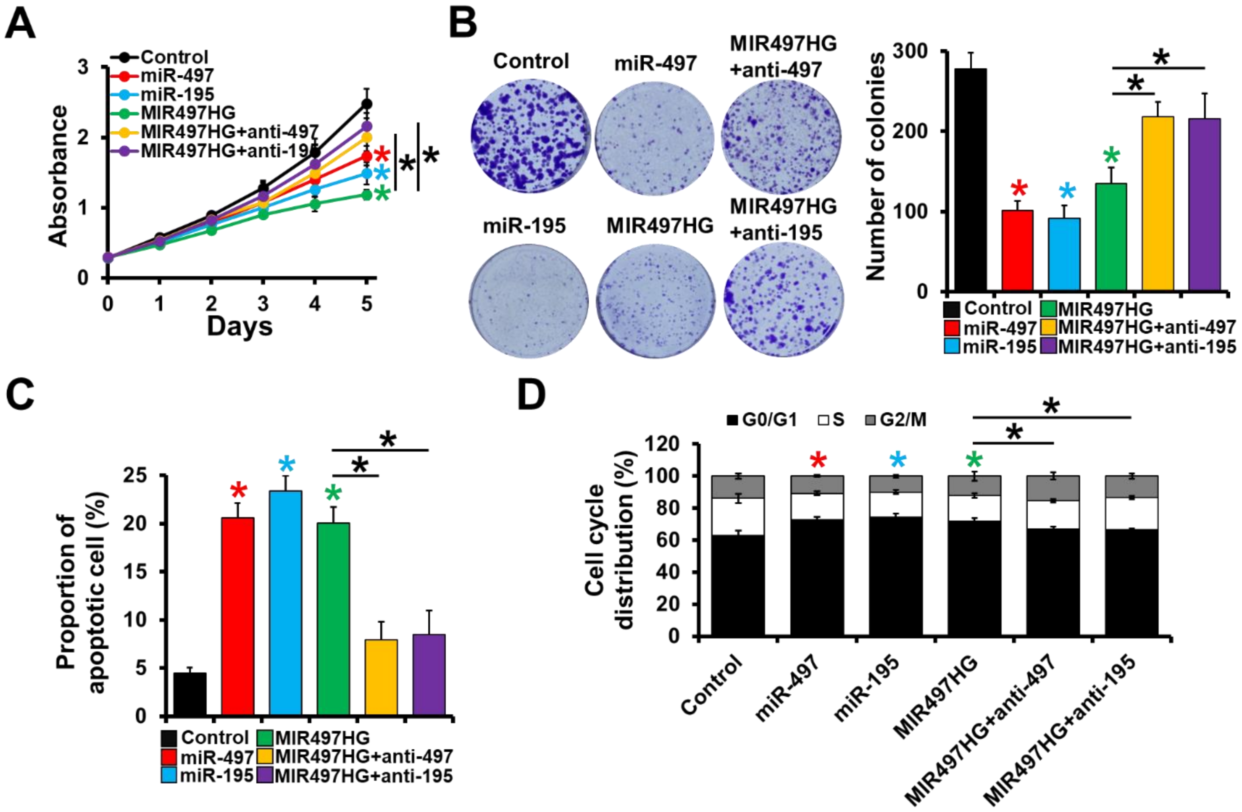

Figure S3. MIR497HG inhibits breast cancer progression by regulating miR-497 and miR-195. A-B, Proliferation abilities of MCF7 cells transfected with MIR497HG plasmid with anti-miR-497 or anti-miR-195, as well as control cells were determined by MTT (A) and colony formation (B) assays. **C,** Cell apoptosis and **D,** cell cycle distribution of MCF7 cells transfected with MIR497HG plasmid with anti-miR-497 or anti-miR-195, as well as control cells were determined by flow cytometry.

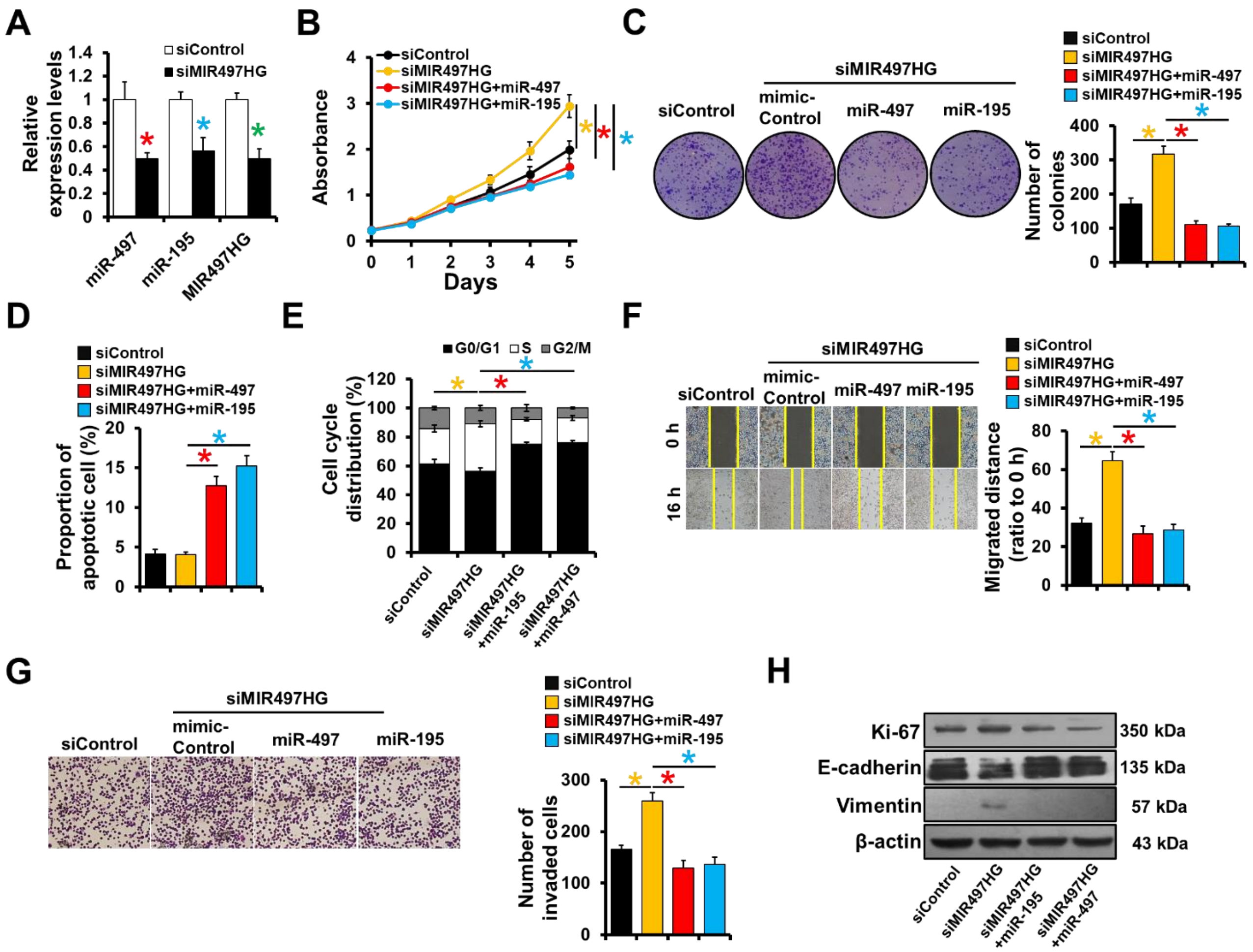

Figure S4. Depletion of MIR497HG promotes breast cancer progression by regulating miR-497/195 expression. A, qRT-PCR results showing levels of MIR497HG and miR-497/195 expression in MIR497HG siRNAs-transfected T47D cells and control cells. **B-C,** Proliferation abilities of T47D cells transfected with siRNAs targeting MIR497HG with miR-497 or miR-195 mimics, as well as control cells were determined by MTT (B) and colony formation (C) assays. **D,** Cell apoptosis and **E,** cell cycle distribution of T47D cells transfected with siRNAs targeting MIR497HG with miR-497 or miR-195 mimics, as well as control cells were determined by flow cytometry. **F,** Migration abilities of T47D cells with different transfections were analyzed by wound-healing assay. **G,** Invasion abilities of T47D cells with different transfections were determined by Transwell assays. **H,** Western blots showing expression levels of epithelial marker E-cadherin, mesenchymal marker Vimentin and Ki-67 proteins in T47D cells expressing MIR497HG siRNAs with miR-497 or miR-195 mimics, as well as control cells. *$P < 0.05$.

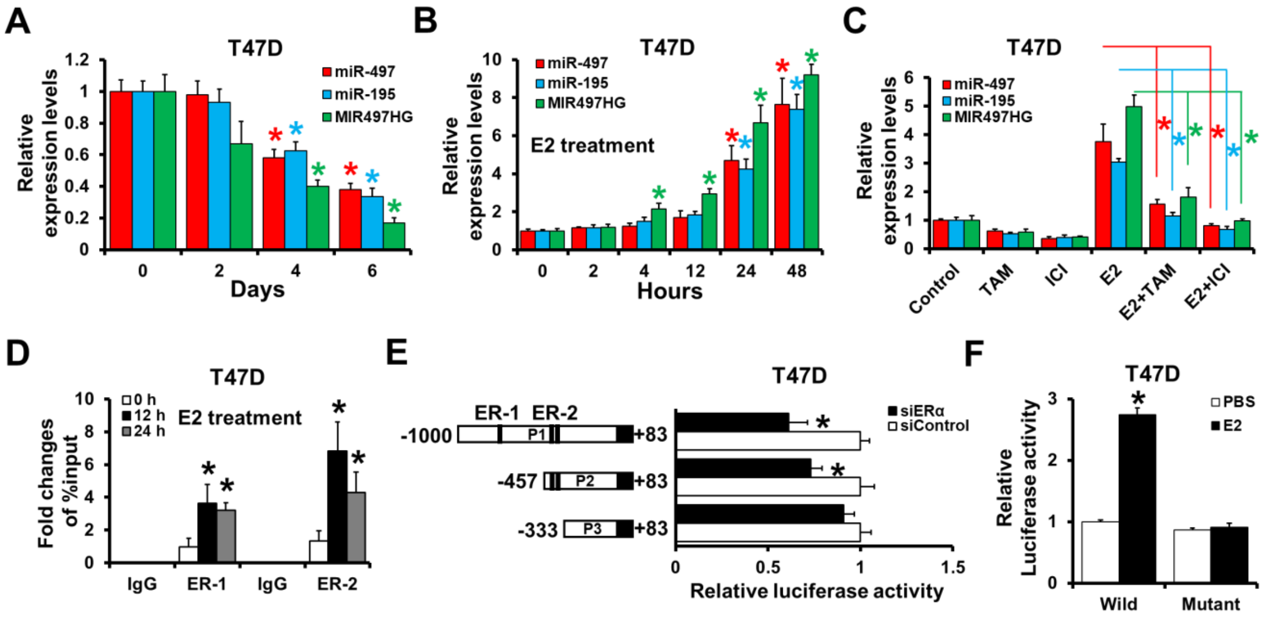

Figure S5. ERα transactivates the MIR497HG expression. A, qRT-PCR results showing levels of MIR497HG and miR-497/195 expression in hormone-deprived T47D cells. **B,** qRT-PCR results showing levels of MIR497HG expression in hormone-deprived T47D cells after treatment with E2 (10 nM). **C,** Levels of MIR497HG and miR-497/195 expression in hormone-deprived T47D cells after 48 h of treatment with E2 (10 nM) and/or tamoxifen/fulvestrant (1 μM). **D,** Enrichment of ERα on the MIR497HG promoter region in hormone-deprived T47D cells determined by ChIP sequence analysis. T47D cells were subsequently treated with 10 nM E2 for 12 or 24 h. Enrichment of MIR497HG promoter DNA in the ERα-immunoprecipitated samples was subsequently determined by qPCR. **E,** Dual-luciferase reporter assay results showing regulation of MIR497HG promoter activity by ERα. Several luciferase reporter plasmids, containing different deleted MIR497HG promoter regions, were transfected with ERα siRNAs into T47D cells. **F,** Regulation of ERα-binding site-mutated MIR497HG promoter activity by E2 as determined by dual-luciferase reporter assay. T47D cells cultured in hormone-deprived condition were transfected with wild-type or mutant MIR497HG promoter luciferase reporter plasmids, then treated with 10 nM E2 for 48 h. *$p < 0.05$.

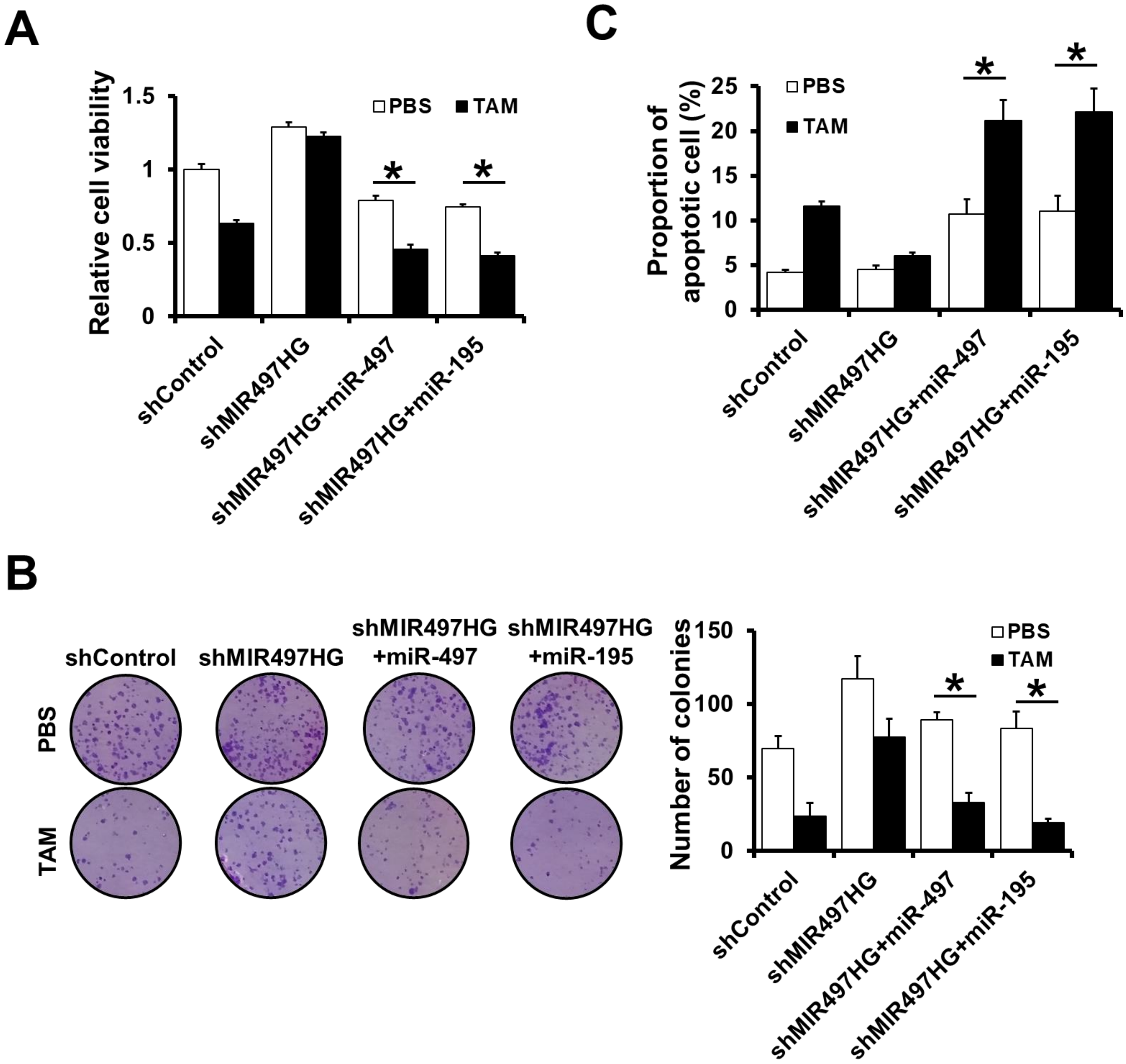

Figure S6. MIR497HG is involved in tamoxifen resistance by regulating miR-497/195 expression. A and B, Proliferation abilities of MIR497HG-depleted MCF7 cells transfected with miR-497 or miR-195 mimics, as well as control cells with or without 1 μM tamoxifen treatment were determined *via* MTT (A) and colony formation (B) assays. **C,** Apoptosis of MIR497HG-depleted MCF7 cells transfected with miR-497 or miR-195 mimics, as well as control cells with or without 1 μM tamoxifen treatment were determined by flow cytometry. *$P < 0.05$.

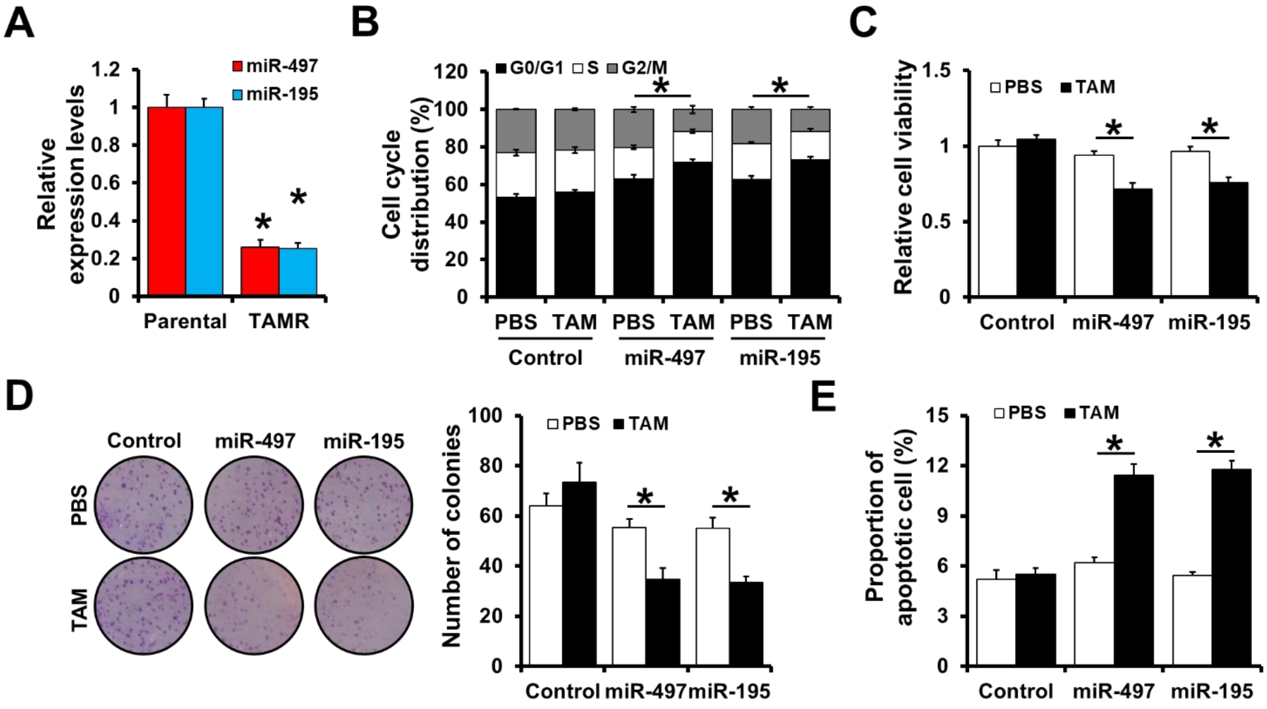

Figure S7. Overexpression of miR-497/195 enhances tamoxifen sensitivity in ER+ breast cancer cells. A, RT-qPCR results showing levels of miR-497/195 expression in MCF7 parental and TamR cells. **B,** Cell cycle distribution of miR-497- or miR-195-overexpressed MCF7/TamR and control cells with or without 1 μM tamoxifen treatment was examined by flow cytometry. **C and D,** Proliferation abilities of miR-497- or miR-195-overexpressed MCF7/TamR and control cells with or without 1 μM tamoxifen treatment were examined by MTT (C) and colony formation (D) assays. **E,** Cell apoptosis of miR-497- or miR-195-overexpressed MCF7/TamR and control cells with or without 1 μM tamoxifen treatment was determined by flow cytometry. *$p < 0.05$.

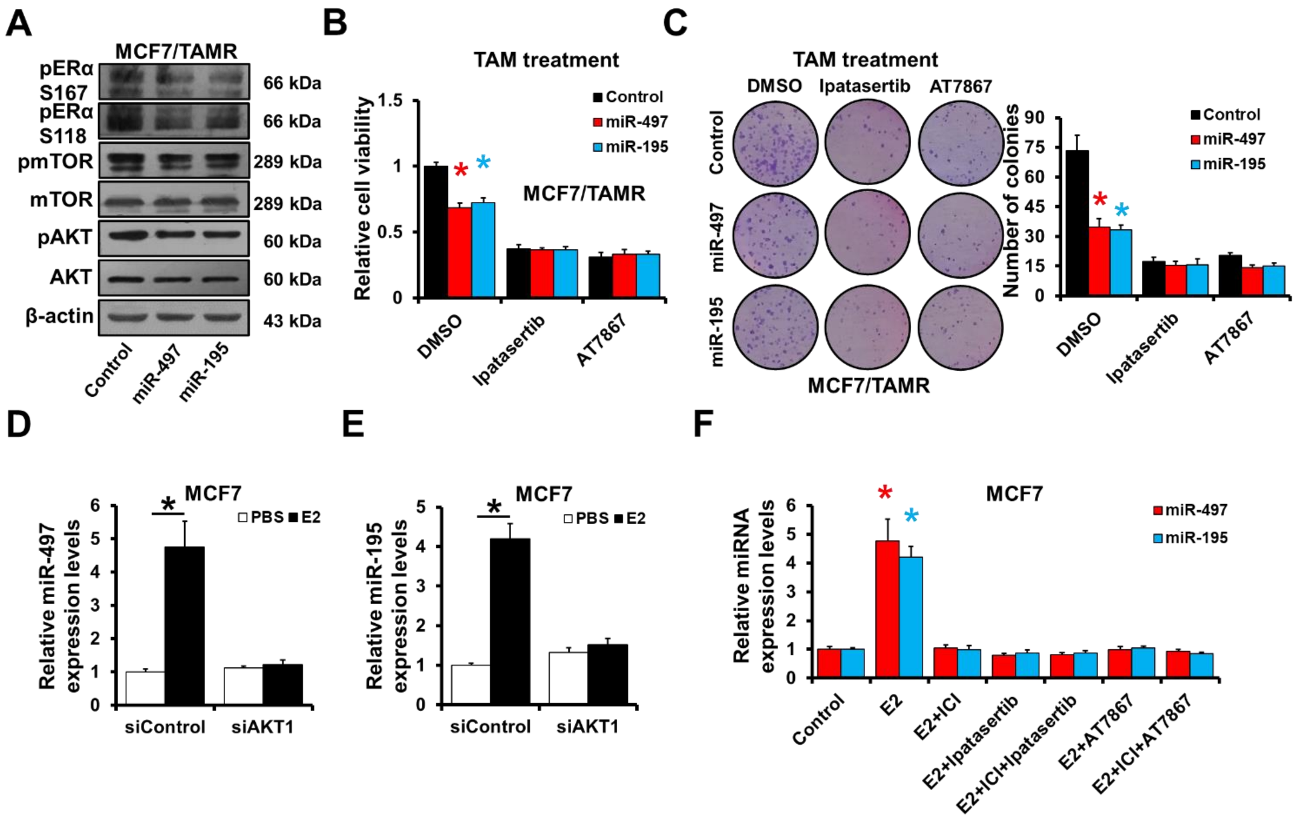

Figure S8. miR-497/195 regulates tamoxifen sensitivity through PI3K-Akt signaling. A, Western blots showing expression of phosphorylated and total Akt, mTOR and ERα in miR-497- or miR-195-overexpressed MCF7/TamR and control cells. **B and C,** Proliferation abilities of miR-497- or miR-195-overexpressed MCF7/TamR and control cells with Ipatasertib or AT7867 treatment were determined by MTT (B) and colony formation (C) assays. **D and E,** RT-qPCR results showing levels of miR-497 (D) and miR-195 (E) in MCF7 cells transfected with siRNAs targeting AKT1 in the presence or absence of 10 nM E2. **F,** RT-qPCR results showing levels of miR-497 and miR-195 in indicated MCF7 cells. *$P < 0.05$.

Table S1. Oligonucleotides of miRNAs and siRNAs

mimic control	UGUACCAAUUUCCAGUGGAGAU
inhibitor control	AUGGUGUUAUCAAGUGUAACAG
miR-497 mimics	UAUGCAUUGUAUUUUUAGGUCC
miR-497 inhibitor	AUACGUAACAUAAAAAUCCAGG
miR-195 mimics	UAGCAGCACAGAAAUAUUGGC
miR-195 inhibitor	GCCAAUAUUUCUGUGCUGCUA
siERα	GCCUGGUCAGAUUACGUAUGC
siZEB1	GCUGUUGUUCUGCCAACAGTT
siAKT1	GCACCUUCAUUGGCUACAATT
siDNMT1	GGAGAACGGUGCUCAUGCUU
siDNMT3B	AGAUGACGGAUGCCUAGAG
siHDAC1	CCGGUCAUGUCCAAAGUAA
siHDAC2	CGGUUAGGUUGCUUCAAUCUA

Table S2. Oligonucleotides used for RT-qPCR

Name	Sequence (5' to 3')
MIR497HG up<br>MIR497HG low	AATGTGAGGGATGCACCCTA<br>CTGTTCCAGACAGCCTGACC
miR-497 up<br>miR-497 low	CAGCAGCACTGTGGTTTGT<br>CGACAGCAGCACACTGTGGTT
miR-195 up<br>miR-195 low	ACACTCCAGCTGGGTAGCAGCACAGAAAT<br>TGGTGTCGTGGAGTCG
U6 up<br>U6 low	CTCGCTTCGGCAGCACA<br>AACGCTTCACG AATTTGCGT
AKT3 up<br>AKT3 low	TGTGTACCGTGATCTCAAGTTGG<br>GAATGTCTTCATGGT GGCTGC
BCL2 up<br>BCL2 low	CAGAATATCAGCCACCTCTT<br>CAGAATATCAGCCACCTCTT
MAP2K1 up<br>MAP2K1 low	CCAAAATGCCCAAGAAGAAGCCG<br>CCAAACACTTAGACGCCAGCAGC
RAF1 up<br>RAF1 low	AGACTGCTCACAGGGCCTTA<br>CTGCAAATGGCTTCCTTCTC
CCND1 up<br>CCND1 low	GGGTTGTGCTACAGATGATAGAG<br>AGACGCCTCCTTTGTGTTAAT
ZEB1 up	TCAAAAGGAAGTCAATGGACAA

ZEB1 low	GTGCAGGAGGGACCTCTTTA
GAPDH up<br>GAPDH low	CAAGGTCATCCATGACAACTTTG<br>GTCCACCACCCTGTTGCTGTAG

Table S3. Antibodies used for study

Name	Source	Catalog
DNMT1 (D63A6) XP® Rabbit mAb	Cell Signaling	5032
DNMT3A (D2H4B) Rabbit mAb	Cell Signaling	32578
DNMT3B (E8A8A) XP® Rabbit mAb	Cell Signaling	57868
β-Actin (8H10D10) Mouse mAb	Cell Signaling	3700
Estrogen Receptor α (D8H8) Rabbit mAb	Cell Signaling	8644
ERα (phospho Ser167) Polyclonal Antibody	Immunoway	YP0105
ERα (phospho Ser118) Polyclonal Antibody	Immunoway	YP0104
mTOR (7C10) Rabbit mAb	Cell Signaling	2983
Phospho-mTOR (Ser2448) (D9C2) XP® Rabbit mAb	Cell Signaling	5536
Akt (5G3) Mouse mAb	Cell Signaling	2966
Phospho-Akt (Ser473) (D9E) XP® Rabbit mAb	Cell Signaling	4060
MEK1 (61B12) Mouse mAb	Cell Signaling	2352
AKT3 Antibody	Cell Signaling	4059
Bcl-2 (124) Mouse mAb	Cell Signaling	15071
c-Raf (D4B3J) Rabbit mAb	Cell Signaling	53745
Cyclin D1 (E3P5S) XP® Rabbit mAb	Cell Signaling	55506
Ki-67 Monoclonal Antibody	Immunoway	YM0391
E-cadherin antibody (1.B.54)	SantaCruz Biotech	sc-71009
Vimentin antibody (V9)	SantaCruz Biotech	sc-6260
Anti-ZEB1 antibody	Abcam	ab180905
HDAC1 (D5C6U) XP® Rabbit mAb	Cell Signaling	34589
HDAC2 (D6S5P) Rabbit mAb	Cell Signaling	57156
Acetyl-Histone H3 (Lys27) (D5E4) XP® Rabbit mAb	Cell Signaling	8173
Acetyl-Histone H4 (Lys5) (D12B3) Rabbit mAb	Cell Signaling	8647

Table S4. Oligonucleotides used for ChIP and methylation specific PCR

Name	Sequence (5’ to 3’)
ER site 1 up<br>ER site 1 low	TGCAAGAATCACCTCCTTGT<br>TGCAAGAATCACCTCCTTGT
ER site 2 up<br>ER site 2 low	CTGCCTGTGCAACAGCAATG<br>TGCTCTGTCCCGATGCCAAT
ZEB1 site 1 up<br>ZEB1 site 1 low	AGCCAGTTGAAGGTTCCACA<br>GAGTCGCAGAATTCCGTAGT
ZEB1 site 2 up<br>ZEB1 site 2 low	TGGTAAGTTTCAGGTATGGG<br>AATGTTCCACCTCAAACTAA
UF site 1 up<br>UR site 1 low	AGGTTTTATATTGTGGTGT<br>CACAAAATTCCATAATTCA
UF site 2 up<br>UR site 2 low	TTGGTTGGGATGTTGGGGGTG<br>CCAACAACTCAAACTCCCA
UF site 3 up<br>UR site 3 low	GGTTTTGGTTGGGTGGGGTATG<br>CAAACAAACTTTAAACAAAAATCC
MF site 1 up<br>MR site 1 low	AGGTTTTATATCGCGGCGT<br>CGCAAAATTCCGTAATTCG
MF site 2 up<br>MR site 2 low	TTGGTCGGGATGTCGGGGGCG<br>CCGACGACTCAAACTCCCG
MF site 3 up<br>MR site 3 low	GGTTTTGGTCGGGCGGGGTACG<br>CAAACGAACTTTAAACGAAAATCC
